# Supplementary figures and images for: Structural analyses of the PKA RIIβ holoenzyme containing the oncogenic DnaJB1-PKAc fusion protein reveal protomer asymmetry and fusion-induced allosteric perturbations in fibrolamellar hepatocellular carcinoma
Source: PLoS Biol. 2020 Dec 28;18(12):e3001018. doi: 10.1371/journal.pbio.3001018 (PMC7793292; doi:10.1371/journal.pbio.3001018)

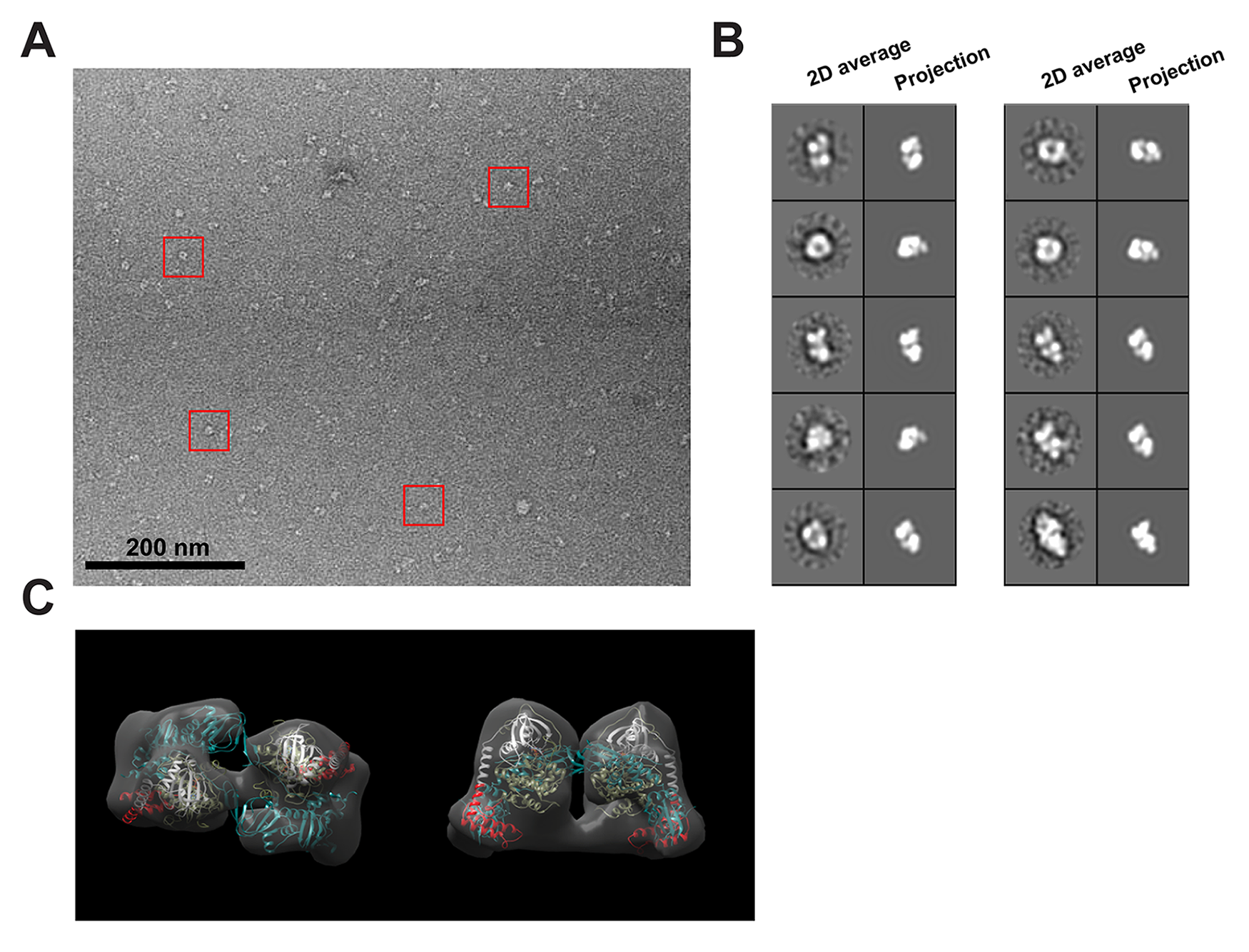

Supplement: S1 Fig — (A) Representative micrograph for negatively stained RIIβ2J-C2 holoenzyme. Example particles are shown in red boxes. (B) 2D class averages of RIIβ2J-C2 shown alongside projections of WT RIIβ holoenzyme crystal structure. The WT RIIβ2C2 crystal structure was filtered to 20Å. (C) Model and negatively stained EM density of RIIβ2J-C2 holoenzyme. EM, electron microscopy; WT, wild-type. (TIF) [file pbio.3001018.s001.tif]

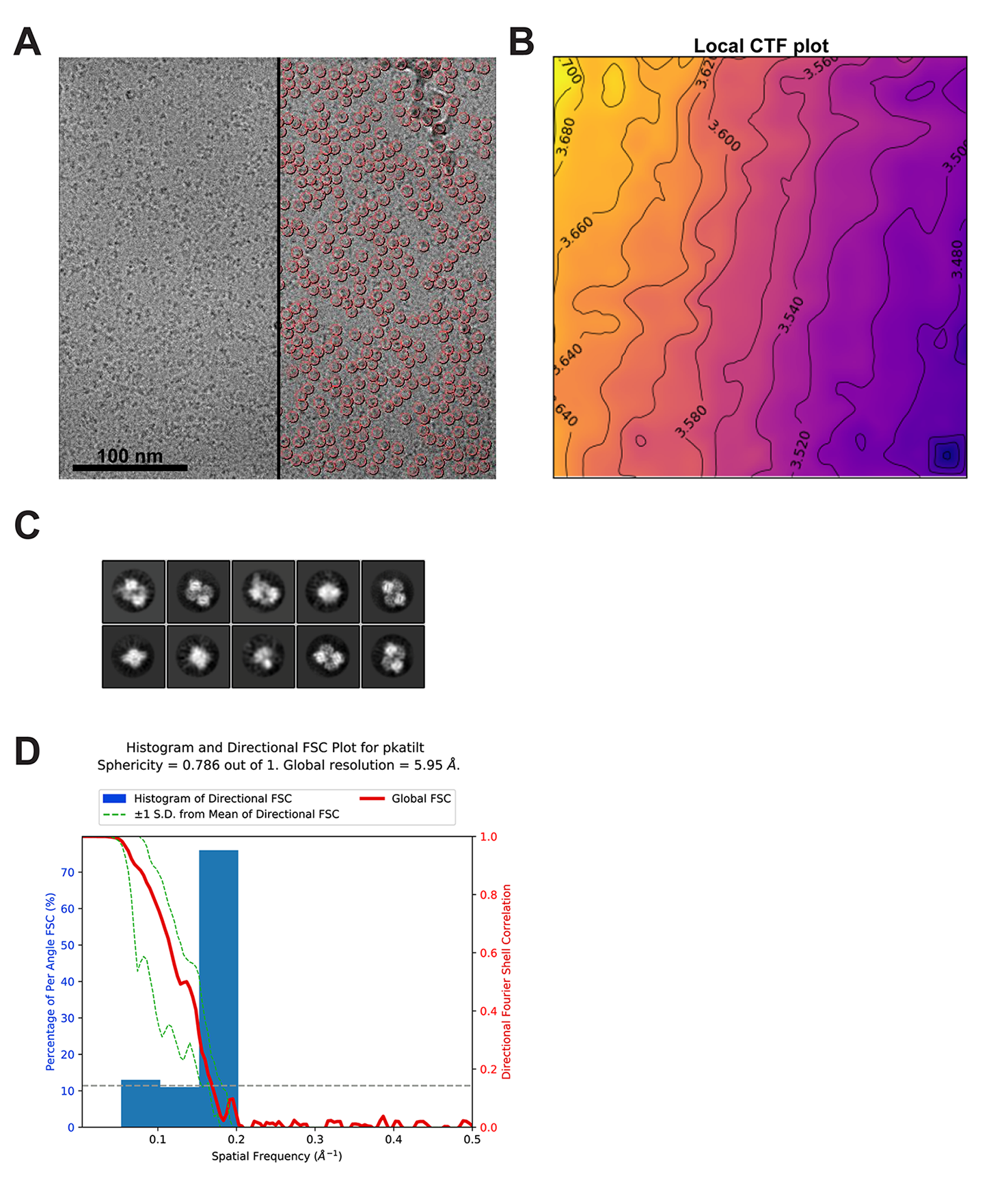

Supplement: S2 Fig — (A) Representative tilted micrograph with the right half of the image showing picked particles. (B) Local CTF plot for micrograph in (A). Image generated using Appion and Gctf. (C) Representative 2D class averages. (D) 3D FSC curve. The data used to make this figure can be found in S1 Data. cryo-EM, cryo-electron microscopy; CTF, contrast transfer function; FSC, fourier shell correlation. (TIF) [file pbio.3001018.s002.tif]

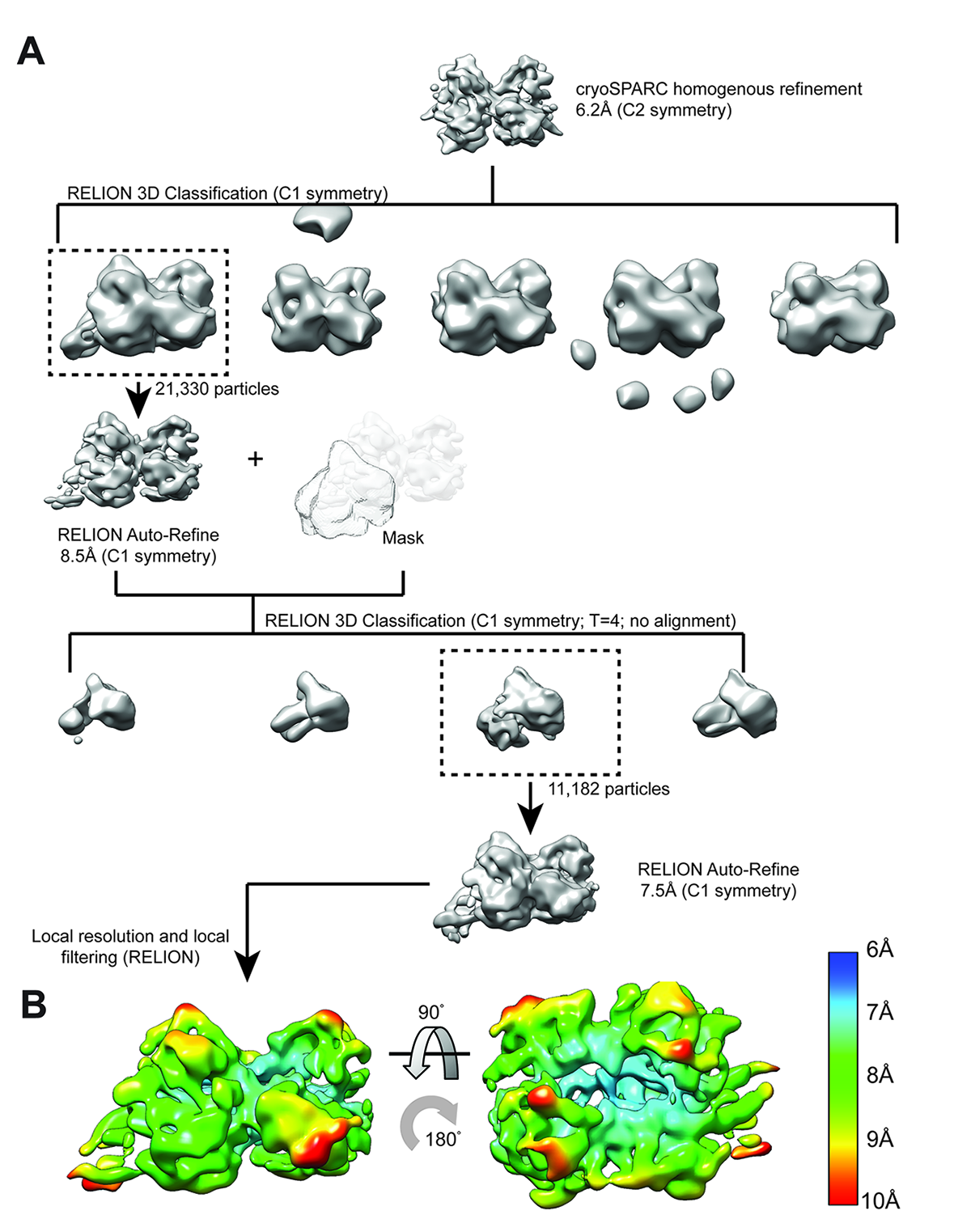

Supplement: S3 Fig — (A) 3D classification. (B) Local density map of RIIβ2J-C2 holoenzyme. (TIF) [file pbio.3001018.s003.tif]

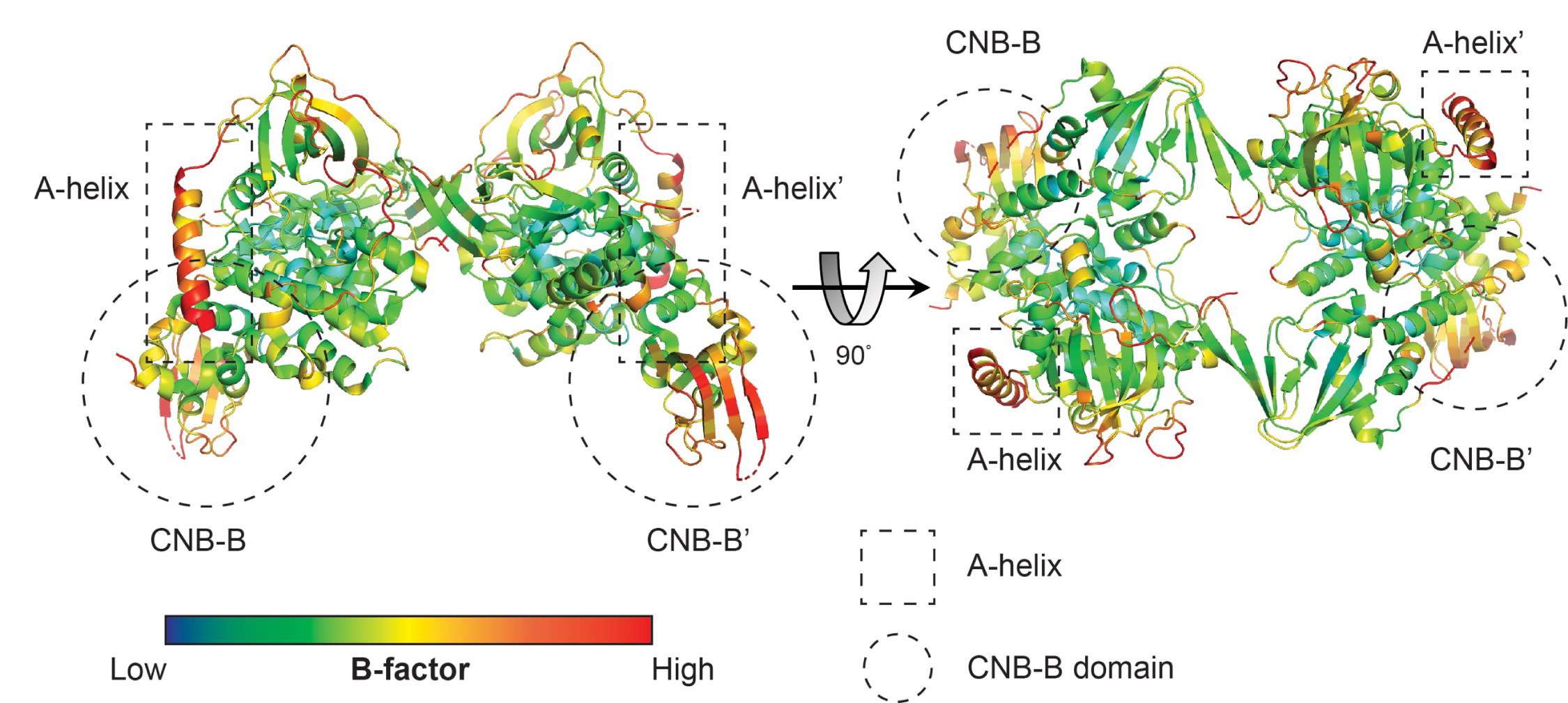

Supplement: S4 Fig — Temperature factor of RIIβ2C2 holoenzyme (PDB = 3TNP). Both the A-helix and the CNB-B domain reveal high temperature factor. PDB, Protein Data Bank. (TIF) [file pbio.3001018.s004.tif]

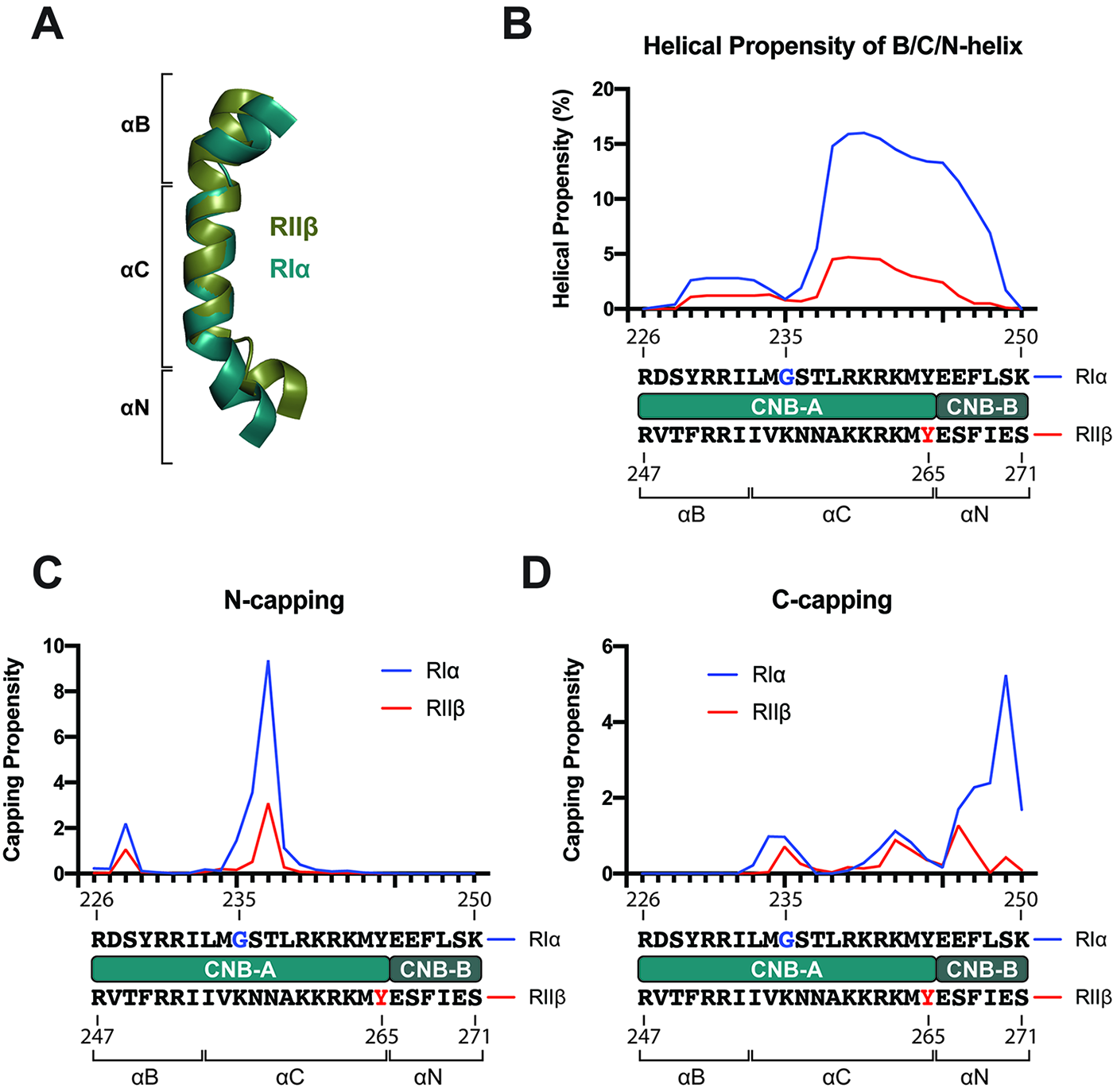

Supplement: S5 Fig — (A) The B/C/N-helices of RIα and RIIβ have different hinge angles. (B) The B/C/N-helix of RIα have high helical propensity with a local minimum at Gly235RIα. In the RIIβ, helical propensity of B/C/N-helix is much lower with a break point at Tyr265RIIβ. (C) N-capping analysis of B/C/N-helix in the RIα and RIIβ. (D) C-capping analysis of B/C/N-helix in the RIα and RIIβ. The data used to make these figures can be found in S1 Data. (TIF) [file pbio.3001018.s005.tif]

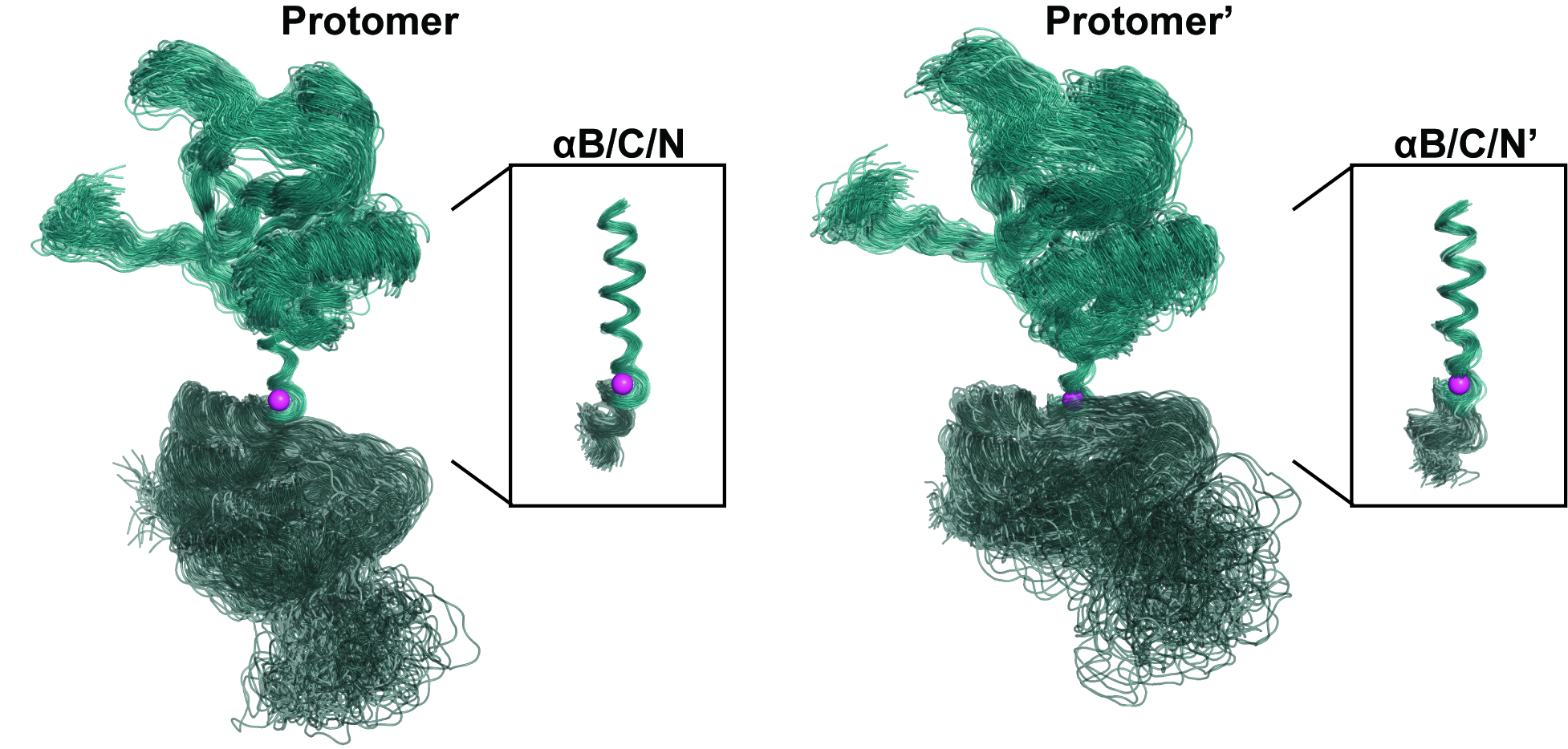

Supplement: S6 Fig — Both CNB domains show similar dynamics and have breakages at Tyr265. Residue Tyr265RIIβ was shown as pink ball. CNB, cyclic nucleotide binding; MD, molecular dynamics. (TIF) [file pbio.3001018.s006.tif]

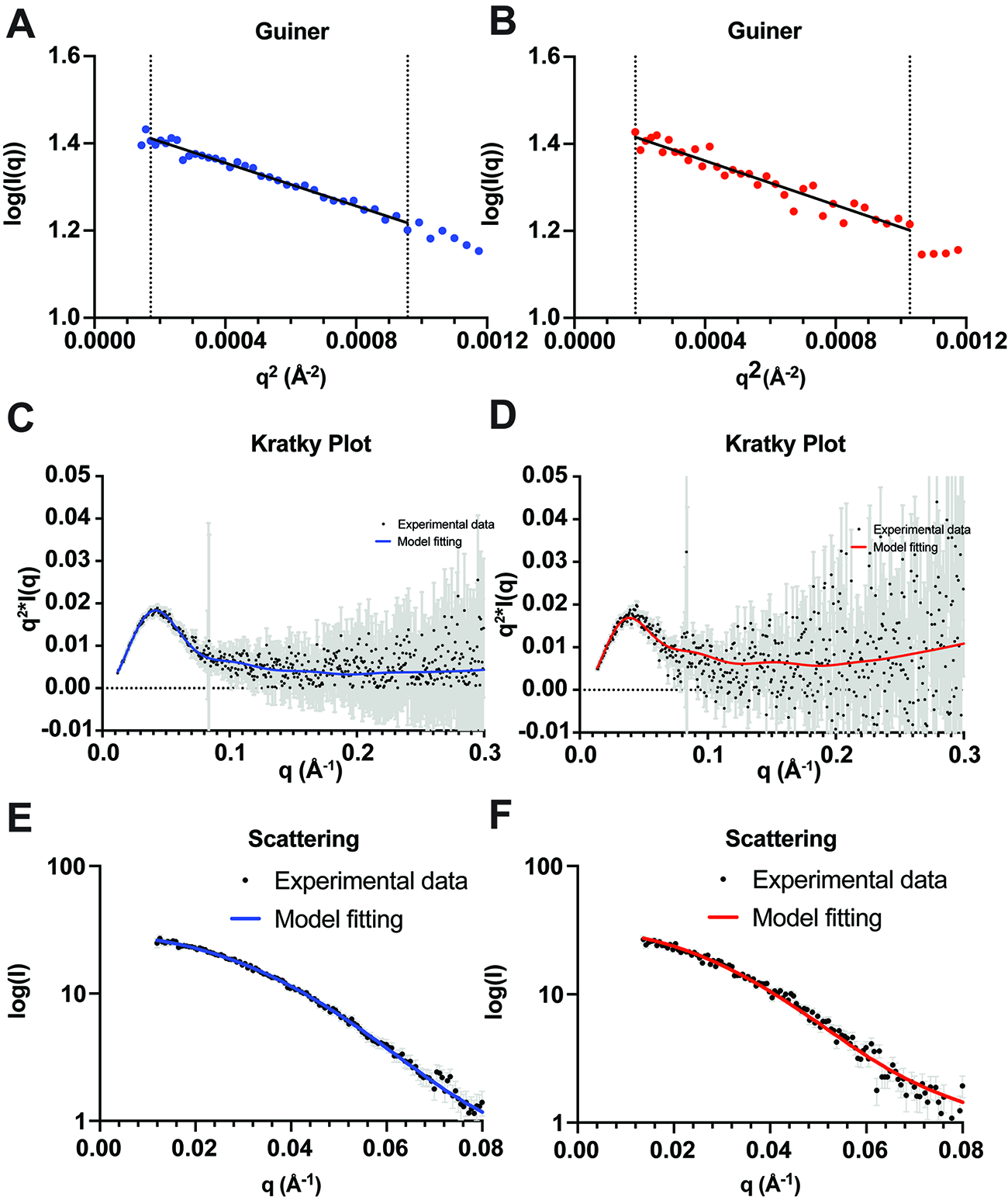

Supplement: S7 Fig — (A, B) Guiner plots of RIIβ2C2 (A) and RIIβ2J-C2 holoenzymes (B). (C, D) Kratky plots of RIIβ2C2 (C) and RIIβ2J-C2 (D) holoenzymes both show bell-shape peaks at low q and not converging to the q-axis at high q. (E, F) Scattering plots at low q and the model fittings of RIIβ2C2 (E) and RIIβ2J-C2 (F) holoenzymes. The data used to make these figures can be found in S1 Data. SAXS, small-angle X-ray scattering. (TIF) [file pbio.3001018.s007.tif]

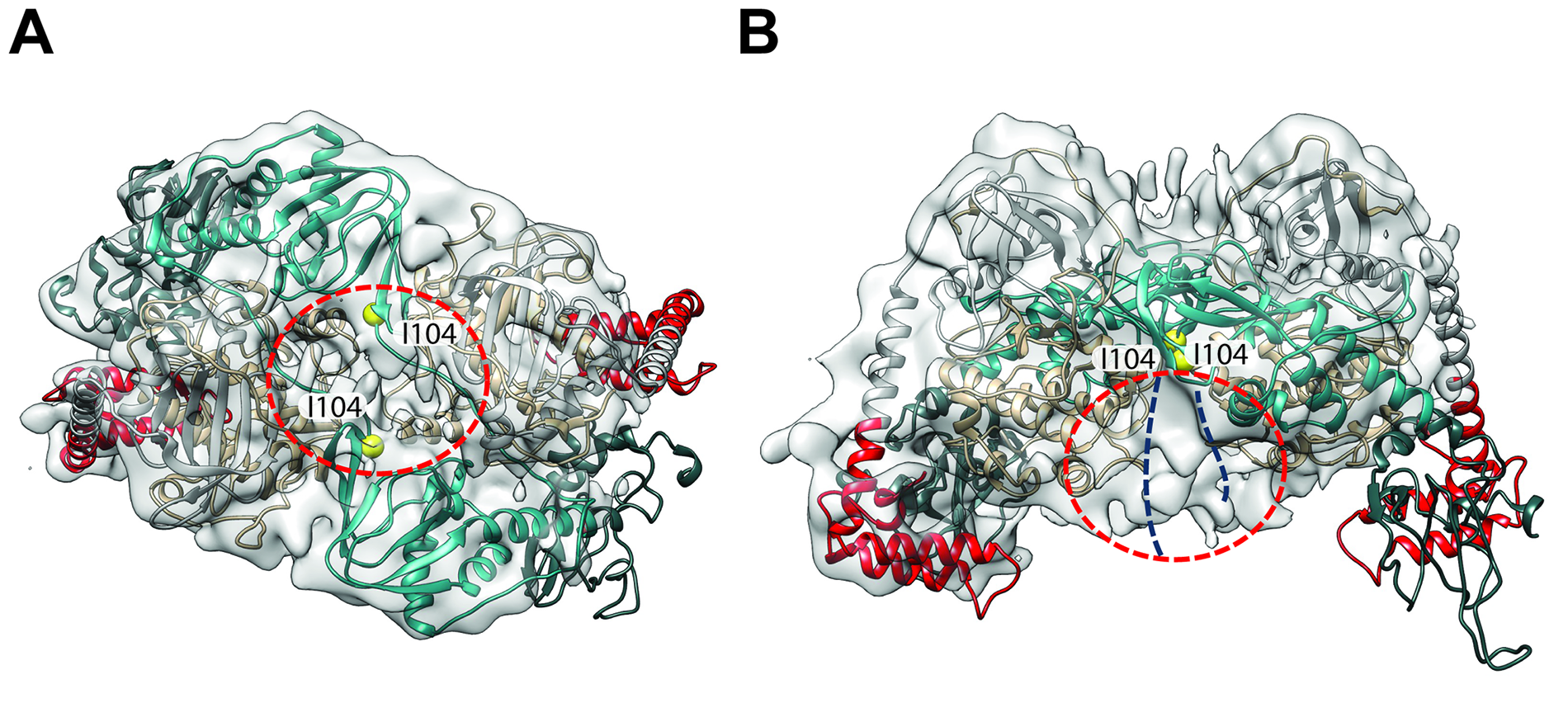

Supplement: S8 Fig — (A) The extra density near the residue Ile104RIIβ locates at the central hole of RIIβ2J-C2 holoenzyme. Residues Ile104RIIβ were labeled as yellow balls. (B) The extra density extends along the central hole to the same face as the CNB-B domains and J-domains. Residues Ile104RIIβ were labeled as yellow balls. CNB, cyclic nucleotide binding; cryo-EM, cryo-electron microscopy; D/D, Dimerization/Docking. (TIF) [file pbio.3001018.s008.tif]

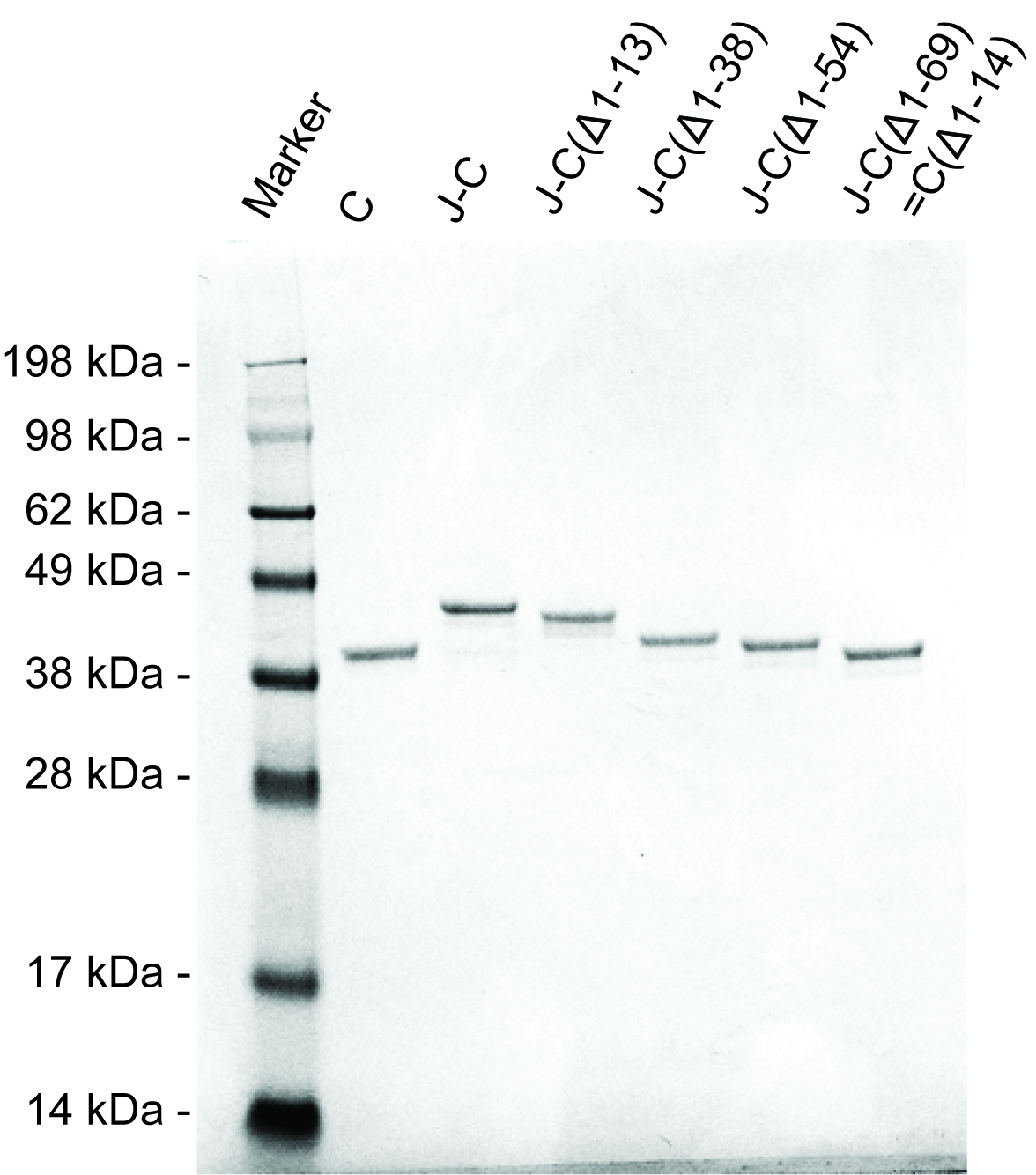

Supplement: S9 Fig — J-C(Δ1–69) subunit is equivalent to C(Δ1–14) subunit. (TIF) [file pbio.3001018.s009.tif]

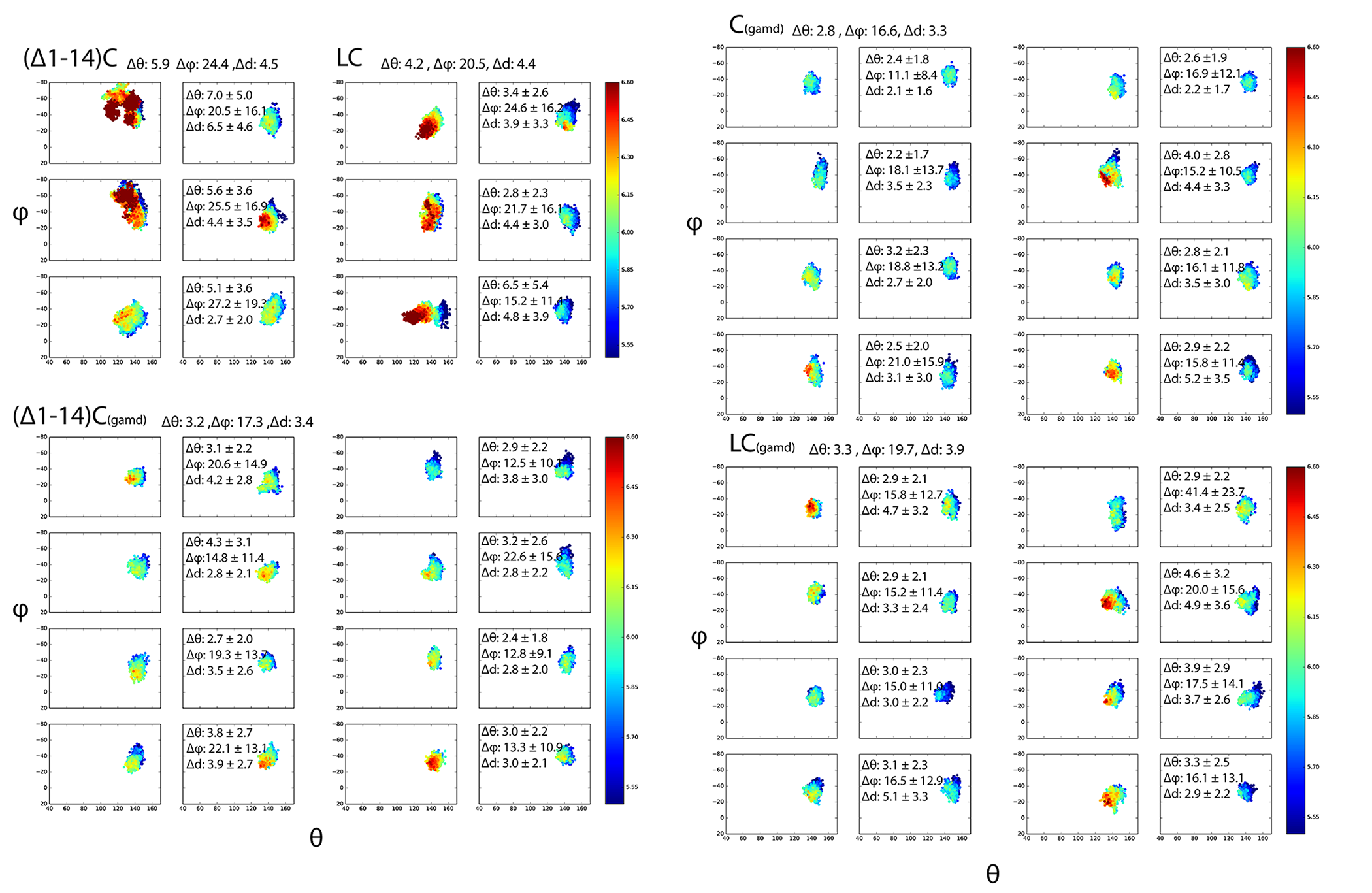

Supplement: S10 Fig — Each pair of panels shows the 2 protomers (left and right) in the holoenzyme. Plots show θ(x), φ(y), and (d) distance in color. Each row is an independent simulation. Upper left panels are conventional MD, as shown in Fig 3 of the main text, and other panels are from GaMD. Full-length C (C-gamd) shows on average a muted asymmetry between protomer displacement vectors. Asymmetry (Δθ,Δφ,Δd) is measured as the average per frame difference of θ, φ, and d between protomers, calculated as ΔX=100*∑i,jN(Xi/Xi¯−Xj/Xj¯)N, where i and j are protomers at matching frames. The data used to make these figures can be found in S1 Data. CNB, cyclic nucleotide binding; GaMD, Gaussian accelerated MD; MD, molecular dynamics. (TIF) [file pbio.3001018.s010.tif]

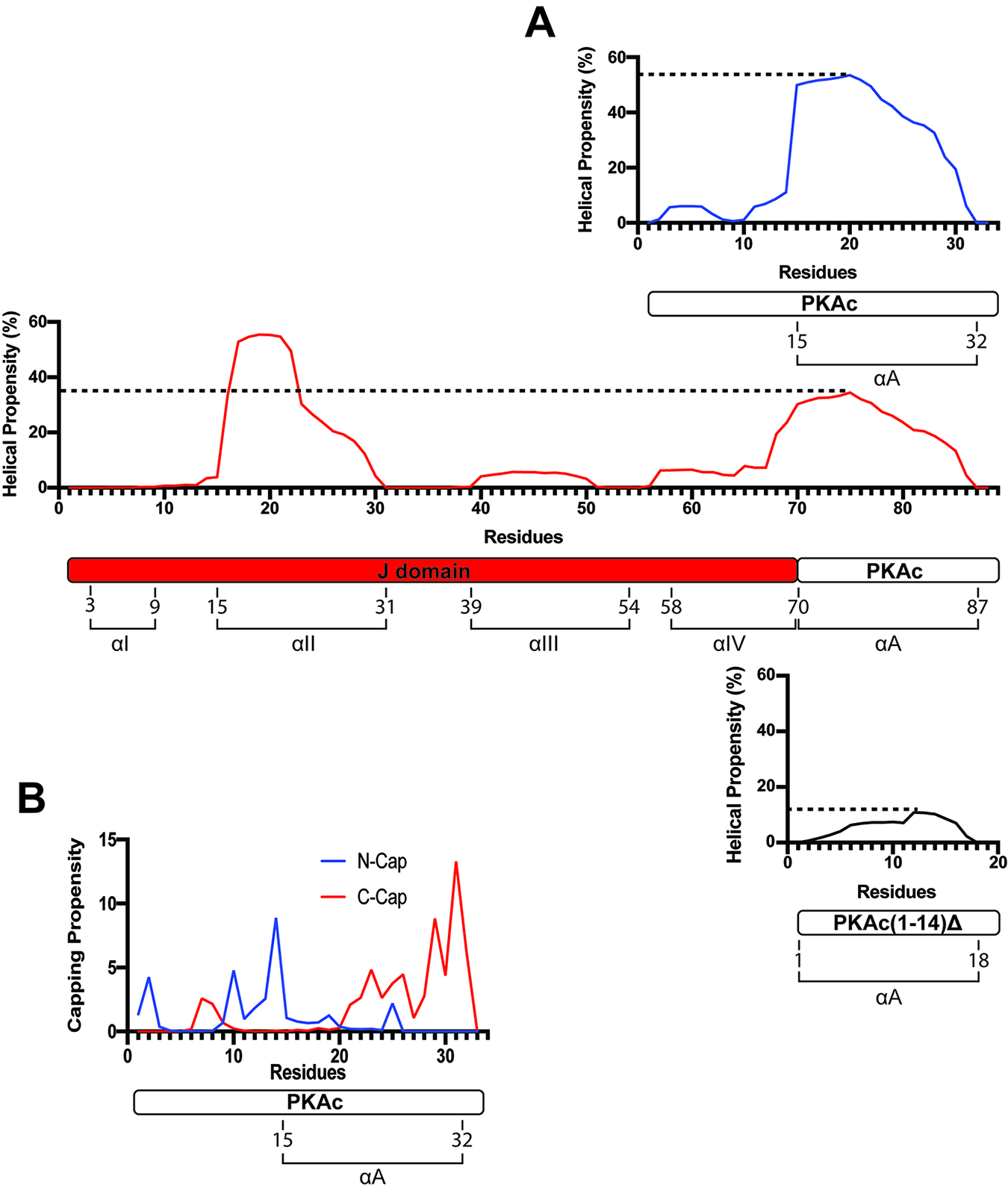

Supplement: S11 Fig — (A) Either fusing with J-domain or just simply deletion of first exon reduces the helical propensity of A-helix. (B) Capping propensity analysis of the A-helix. Ser14C reveals a strong N-capping propensity. The data used to make these figures can be found in S1 Data. (TIF) [file pbio.3001018.s011.tif]

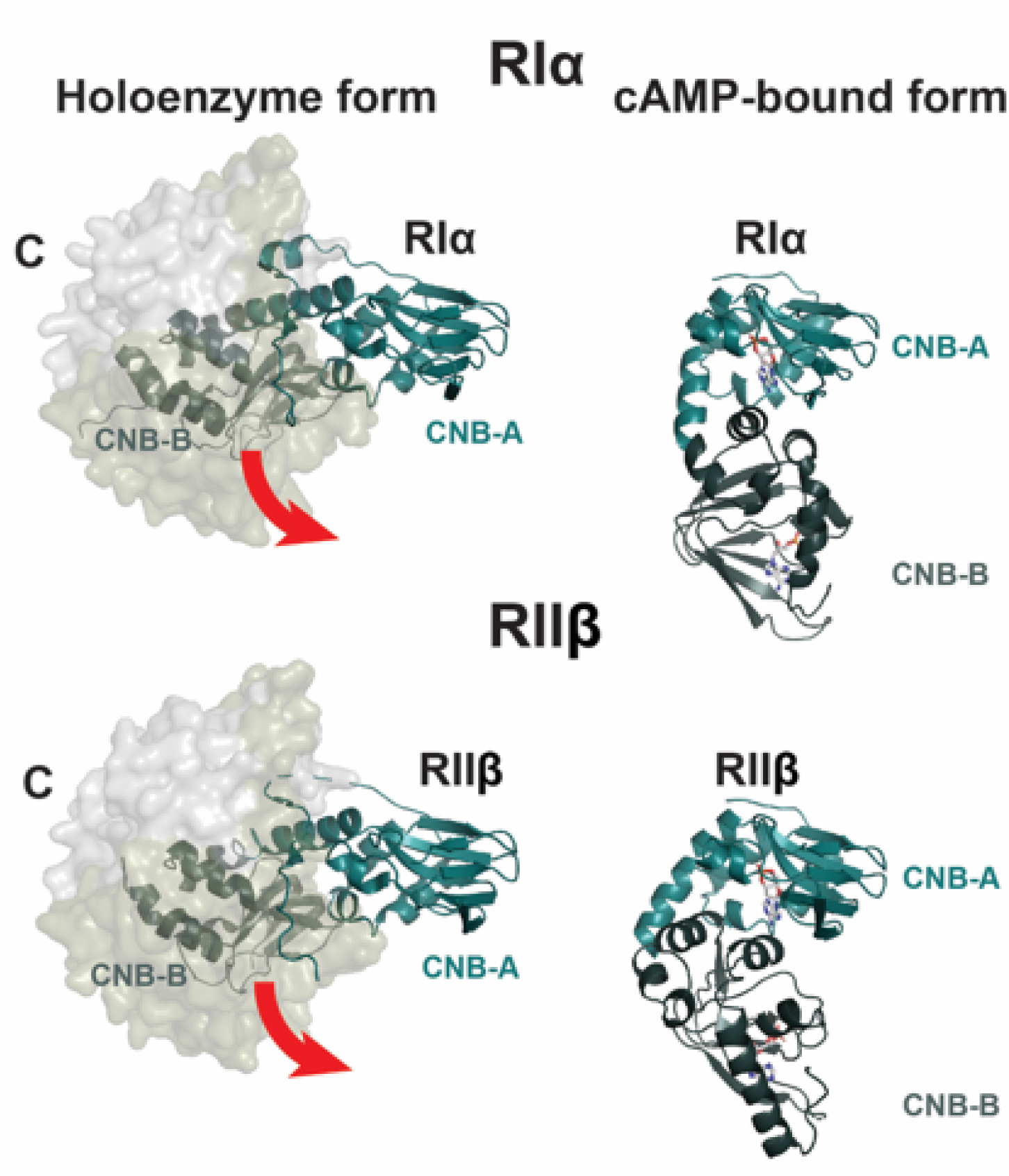

Supplement: S12 Fig — PKA RIα and RIIβ undergo different conformational changes between holoenzyme form and cAMP-bound form. PKA, cAMP-dependent protein kinase. (TIF) [file pbio.3001018.s012.tif]

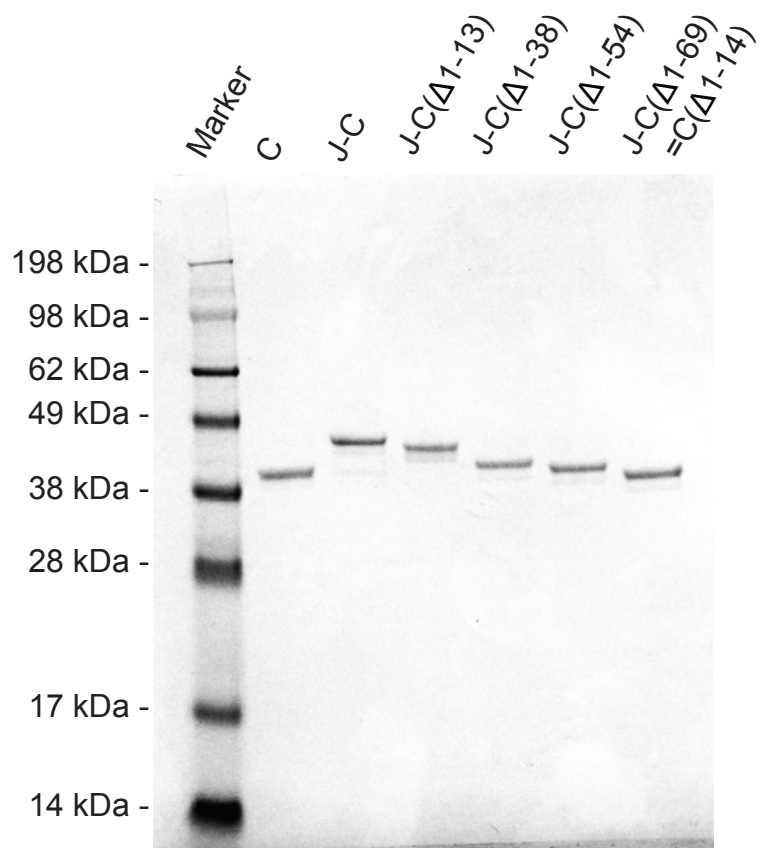

Supplement: S1 Raw Images — J-C(Δ1–69) subunit is equivalent to C(Δ1–14) subunit. (PDF) [file pbio.3001018.s014.pdf]
