# Supplementary material for: Structural analyses of the PKA RIIβ holoenzyme containing the oncogenic DnaJB1-PKAc fusion protein reveal protomer asymmetry and fusion-induced allosteric perturbations in fibrolamellar hepatocellular carcinoma
Source: PLoS Biol. 2020 Dec 28;18(12):e3001018. doi: 10.1371/journal.pbio.3001018 (PMC7793292; doi:10.1371/journal.pbio.3001018)
Supplement: S1 Methods — (DOCX) [file pbio.3001018.s013.docx]

**Materials and Methods**

**Protein purification**

All wild-type C-subunit, J-C-subunit, and deletion mutants of J-C-subunit were purified with a similar purification protocol. The constructs were transformed into BL21(DE3), 0.5 mM IPTG was added to induce the cells at OD_600_=0.6-0.8. After 16 hours of expression at 4°C. The bacterial pellets were re-suspended and lysed in the lysis buffer: 20 mM Tris-Cl, 300 mM NaCl, 5 mM β-mercaptoethanol (BME). After high speed centrifugation (13,000 rpm, 1hr), the supernatant was collected then passed through the Ni-resin. The resin was first washed with 3CV of wash buffer (20 mM Tris-Cl pH=8.0, 300 mM NaCl, 10 mM imidazole, 5 mM BME), and then C-subunit was eluted by 3CV of elution buffer (20 mM Tris-Cl pH=8.0, 300 mM NaCl, 500 mM imidazole, 5 mM BME). The eluent was collected and supplemented with Ulp1 (molar ratio SUMO-C : Ulp1 = 200 : 1). The mixture was dialyzed against the buffer (20 mM Tris-Cl pH=8.0, 300 mM NaCl, 5 mM BME) at 4°C overnight. After SUMO tag cleavage, the cleaved SUMO tag and uncleaved protein were removed by passing the solution back through the Ni-resin. The proteins were further purified by S-75 gel filtration column with buffer (20 mM MES pH=6.5, 300 mM NaCl, 5 mM BME).

RIIβ-subunit purifications are similar same as the literatures[1, 2].

**Holoenzyme formation**

Holoenzyme was prepared by mixing RIIβ-subunit (1 equiv.) with excess of C-subunits (1.3 equiv.). The mixture was dialyzed against holoenzyme buffer: 50 mM MES pH=5.8, 50 mM NaCl, 1mM TCEP. Unbound C subunits was removed by S-200 gel filtration column with the same holoenzyme buffer.

**Grid preparation and data acquisition**

For negative stain sample preparation, 4 µL of RIIβ_2_J-C_2_ at 40nM was incubated on continuous carbon grids (Electron Microscopy Sciences, Hatfield, PA; CF400-Cu) for 30 s. After incubation, the grid was transferred directly onto 5 × 75 µl droplets of 2% uranyl acetate and then blotted dry. Negative stain grids were imaged on an Tecnai Sphera (FEI Company, Hillsboro, OR) at 200 kV using a US4000 CCD detector (Gatan Inc., Pleaston, CA) with the Leginon automated data collection software[3] over a defocus range of 1–2.5 µm and a dose of 40 e-/Å^2^ at a nominal magnification of 62,000X (1.90 Å/pix).

For cryo-EM sample preparation, 4µl of RIIβ_2_J-C_2_ at 2µM was applied to a glow-discharged UltraAuFoil R (1.2/1.3) 300-mesh gold grid (Electron Microscopy Sciences). The grids were then blotted with filter paper and plunge-frozen into ethane cooled with liquid nitrogen using a Vitrobot Mark IV (Thermo Fisher Scientific) set to 4 °C, 100% humidity, 4 s blot, and a force of 20. Micrographs were collected using Leginon[3] on a Titan Krios transmission electron microscope (Thermo Fisher Scientific) operating at 300 keV using a Gatan K2 Summit direct electron detector (Gatan, Inc) in counting mode (1 Å/pixel) at a nominal magnification of 29,000X using 100 ms frame rate for 10 seconds with a total dose ~78e/Å^2^. All images were collected using a stage tilt of 40 degrees to increase orientation distribution [4, 5]. Appion[6] was used for on-the-fly preprocessing to run MotionCor2[7] and CTFFIND4[8].

**Image processing**

Particles were picked and extracted using Appion preprocessing software. 693,293 particles were picked from 1,129 micrographs with the FindEM[9] software, using 2D class averages from untilted cryo-EM data for RIIβ_2_J-C_2_ as templates (SI Fig. 2A). Particles were extracted using RELION[10] and local CTF values calculated by Gctf[11] (SI Fig. 2B) to produce a particle stack at 1Å/pixel and a box size of 256 pixels. The particle stack was imported into cryoSPARC[12] (v0.65) for 2D classification (SI Fig. 2C). After throwing away bad classes, we used 153,426 particles for ab initio classification into 3 classes using cryoSPARC[12] (v0.65). Although all particles were compact and globular, we selected a single good class containing 69,605 particles for homogenous refinement in cryoSPARC[12] (v0.65), applying C2 symmetry to obtain a 6.2Å structure (-302Å^2^ B-factor). Using the resulting particle stack, we performed 3D classification using RELION (v2.1) into 5 classes (SI Fig. 3A). After selecting the 21,330 particles associated with the map containing an extra density for CNB-B, we performed another round of 3D refinement using RELION to obtain a structure at 8.5Å using C1 symmetry (SI Fig. 3A). In order to obtain more information about the CNB-B domain, we created a mask and used this to perform classification without alignment to enrich for particles containing extra density. From one class, we used 11,182 particles to obtain a 7.5Å structure (C1 symmetry). For this, we then filtered according to local resolution using a B-factor of -500Å^2^ (SI Fig. 3B).

**Model building and refinement**

The crystal structure of WT RIIβ holoenzyme (PDB = 3TNP) and DnaJB1-PKAc (PDB = 4WB7) alone were chosen as initial models. To generate RIIβ_2_J-C_2_ model, the C-subunits in RIIβ holoenzyme were replaced with the J-C-subunits and submitted to HHpred (<https://toolkit.tuebingen.mpg.de/#/tools/hhpred>), using the top 10 scoring homologous models. This model was then initially refined using Rosetta[13] with the locally filtered cryo-EM map. We calculated 200 models and used top scoring model for a final refinement.

The real-space refinement of C1 symmetry structure was carried out by using Phenix. Further model refinement was performed by using Coot[14]. The C2 symmetry structure was derived from the refined C1 symmetry structure. Based on the C2 symmetry map, the N-terminus (residue 1 - 48) of J-C-subunit and CNB-B domain (residue 266 - 416) of RIIβ-subunit were removed from C1 symmetry structure. Both the two structure models have good geometry as evaluated with PROCHECK[15].

**Molecular cloning**

The deletion mutants of J-C-subunit were made by cloned using the Gibson Assembly method. The gene and vector segments were cloned with the pET-His6-SUMO-TEV-DnaJB1-PKAc as template. The DNA segments were assembled using the NEBuilder HiFi DNA Assembly Kit following their suggested protocol. The DNA segments were cloned by using the following primer sets.

| Vector | 5’-ACCACCAATCTGTTCTCTGTG-3’ 5’ -GGAGTTTTCTGAGTTTTAGTAATGAATTGGAAG-3’ |
| --- | --- |
| J-C(Δ1-13) | 5’-GAGAACAGATTGGTGGTGCGTCGGACGAGGAGATCAA-3’ 5’- CTTCCAATTCATTACTAAAACTCAGAAAACTCC-3’ |
| J-C(Δ1-38) | 5’-GAGAACAGATTGGTGGTGGCGCCGAGGAGAAGTTC-3’ 5’-CTTCCAATTCATTACTAAAACTCAGAAAACTCC-3’ |
| J-C(Δ1-54) | 5’-GAGAACAGATTGGTGGTAGCGACCCGCGCAA-3’ 5’-CTTCCAATTCATTACTAAAACTCAGAAAACTCC-3’ |
| J-C(Δ1-69) | 5’-GAGAACAGATTGGTGGTGTGAAAGAATTCTTAGCCAAAGCC-3’ 5’-CTTCCAATTCATTACTAAAACTCAGAAAACTCC-3’ |

**System preparation** **for MD**

Holoenzyme complexes were prepared from either RIIβ_2_C_2_ or RIIβ_2_J-C_2_. The models were processed in Maestro (Schrodinger) where missing sidechains, counter ions, and ioniziable side chains were modeled in the Protein Preparation Wizard. Hydrogens were added and the models were solvated in a cubic box of TIP4P-EW water [16] and 150 mM KCl with a 10 Å buffer in AMBERtools [17]. Parameters from the Bryce AMBER Parameter Database were used for phosphothreonine [18], and phosphoserine [18].

**MD simulations**

AMBER16 [17] was used for energy minimization, heating, and equilibration, using the CPU code for minimization and heating and GPU code for equilibration. 500 steps of hydrogen-only minimization was followed by 500 steps of solvent minimization, 500 steps of sidechain minimization, and 5000 steps of all-atom minimization. Systems were heated from 0K to 300 K over 500 ps with 2 fs timesteps and 10.0 kcal⋅mol⋅Å position restraints on protein and ligand. Temperature was maintained by the Langevin thermostat. Constant pressure equilibration with a 10 Å non-bonded cut-off with particle mesh Ewald was performed with 100 ps of protein and ligand restraints followed by 100 ps without restraints. Hydrogen mass repartition was implemented to achieve a 4 fs time-step for production runs [19]. Production simulations were performed on GPU enabled AMBER16 [20, 21] as above in triplicate for a total aggregate simulation time of 1.5 μs for each complex. Gaussian accelerated MD (GaMD) was also used to enhance conformational sampling[22] of the holoenzyme complexes (C, J-C, and J-C(Δ1-69)). Systems were prepared, minimized, heated, and equilibrated identically to the conventional MD simulations. GaMD applies a Gaussian distributed boot to the potential energy surface which accelerated transitions between states while allowing accurate reweighting with cumulant expansions. Both dihedral and total potential acceleration were used simultaneously. Each GaMD simulation was equilibrated for 50ns during which boost potentials were updated every 2ns. For each construct 8 independent replicas of 100ns of GaMD simulation were run in the NVT ensemble.)

**MD analysis**

The first 50 ns of each simulation was removed prior to analysis. Trajectories were aligned to the least dynamic regions of the protein by superposing only Cα atoms with an initial RMSF less than the mean. The displacement vector between CNBA and CNBB was put into the same reference frame for all constructs by aligning the CNBA domain of each R protomer over CA atoms for residues 169-246 to the same reference structure for both protomers prior to calculating the vector between Arg230 and Arg359. Solvent accessible surface area was calculated for residues 302, 321, 323, 339, 348, 350, 351, 352, 359, 360, 361 which form the cyclic-nucleotide binding pocket in the CNB-B domain of RIIB. The Shrake-Rupley method, within MDtraj, with a probe radius of 0.3nm, similar to the radius of a cAMP molecule, was used for the SASA determination per frame. Plotting and analysis were performed in python.

**SAXS analysis and model building**

SAXS data were collected at beamline SIBYLS at Advanced Light Source equipped with Agilent 1260 series HPLC with a Shodex analytical column. Data were collected at 298K with sample to detector distance at 1.5 m and λ=1.03Å. Samples concentration was both ~5 mg/mL, with 60 µL in each loading. Buffer signals were subtracted based on the measurement of averaged background scattering in each sample. The scattering files were transformed into real space pair distance distribution function *P*(r), and R_g_ and D_max_ were also calculated based on the *P*(r) function [23]. Radius of gyration (R_g_) was calculated from consecutive data throughout the FPLC and checked for consistency. All data were then scaled and merged as the final plot. Both samples have no sign of aggregation according to the Guinier analysis at low scattering angles(q). The Data were analyzed by ATSAS 2.8 [24]. Molecular weight estimation of each complex was calculated based on the formula: MW = Porod Volume / 1.7.

SAXS model was built by using the program CAROL[25]. The flexible regions, such as residues 1-14 in C-subunit, residues 122-129, 44-103, 325-336, and 394-416 in RIIβ-subunit, were built as poly-Gly chains. The structural model of D/D domain was generated based on the homologous modeling by using the online program, I-TASSER[26].

The SAXS *ab initio* models were generated by using the program DAMMIN[27]. The models of each holoenzyme were repeated at least three times, and all of the repeats show similar results. The *ab initio* models were further overlaid with the atomic structure models by using the program SUPCOMB[28].

**Fluorescence polarization assay**

The cAMP activation assay of RIIβ holoenzymes was measured by a fluorescence polarization assay[29]. The holoenzyme was formed by mixing C-subunit (or J-C-subunit or J-C deletion mutants) and RIIβ with molar ratio 1:1.2 in buffer: 2 mM HEPES pH=7.0, 75mM KCl, 0.005% Triton X-100, 1mM DTT, 10mM MgCl_2_, and 1mM AMPPNP. A N-terminus fluorescein-labeled 20-residue PKI(5-24) peptide (FAM-IP20) was then added into the reaction. The working concentration of C-subunit was 12nM, FAM-IP20 was 2nM, and RIIβ was 14.4nM. A two-fold serial dilutions of cAMP from 8,000 to 0nM were added to each reaction. The polarization changes were from the FAM-IP20 binding to the C-subunit. Excitation and emission wavelength (485 nm and at 535 nm, respectively) to measure its polarization. The experiments were carried out with a GENios Pro micro-plate reader (Tecan) using black flat-bottom 96-well plates. Each data was repeated at least four times and the data sets were analyzed with Prism 7.

**Helical and capping propensity analysis**

The online program, Agadir, was used to analyzed the helical and capping residues propensity of B/C/N-helix in RIα and RIIβ, A-helix in wt C-subunit, and J-domain[30].

**References**

1. Zhang P, Smith-Nguyen EV, Keshwani MM, Deal MS, Kornev AP, Taylor SS. Structure and allostery of the PKA RIIbeta tetrameric holoenzyme. Science. 2012;335(6069):712-6. Epub 2012/02/11. doi: 10.1126/science.1213979. PubMed PMID: 22323819; PubMed Central PMCID: PMCPMC3985767.

2. Bruystens JG, Wu J, Fortezzo A, Kornev AP, Blumenthal DK, Taylor SS. PKA RIalpha homodimer structure reveals an intermolecular interface with implications for cooperative cAMP binding and Carney complex disease. Structure. 2014;22(1):59-69. Epub 2013/12/10. doi: 10.1016/j.str.2013.10.012. PubMed PMID: 24316401; PubMed Central PMCID: PMCPMC3963464.

3. Suloway C, Pulokas J, Fellmann D, Cheng A, Guerra F, Quispe J, et al. Automated molecular microscopy: the new Leginon system. J Struct Biol. 2005;151(1):41-60. Epub 2005/05/14. doi: 10.1016/j.jsb.2005.03.010. PubMed PMID: 15890530.

4. Tan YZ, Baldwin PR, Davis JH, Williamson JR, Potter CS, Carragher B, et al. Addressing preferred specimen orientation in single-particle cryo-EM through tilting. Nat Methods. 2017;14(8):793-6. Epub 2017/07/04. doi: 10.1038/nmeth.4347. PubMed PMID: 28671674; PubMed Central PMCID: PMCPMC5533649.

5. Su M, Guo EZ, Ding X, Li Y, Tarrasch JT, Brooks CL, 3rd, et al. Mechanism of Vps4 hexamer function revealed by cryo-EM. Sci Adv. 2017;3(4):e1700325. Epub 2017/04/26. doi: 10.1126/sciadv.1700325. PubMed PMID: 28439563; PubMed Central PMCID: PMCPMC5392032.

6. Lander GC, Stagg SM, Voss NR, Cheng A, Fellmann D, Pulokas J, et al. Appion: an integrated, database-driven pipeline to facilitate EM image processing. J Struct Biol. 2009;166(1):95-102. Epub 2009/03/06. doi: 10.1016/j.jsb.2009.01.002. PubMed PMID: 19263523; PubMed Central PMCID: PMCPMC2775544.

7. Zheng SQ, Palovcak E, Armache JP, Verba KA, Cheng Y, Agard DA. MotionCor2: anisotropic correction of beam-induced motion for improved cryo-electron microscopy. Nat Methods. 2017;14(4):331-2. Epub 2017/03/03. doi: 10.1038/nmeth.4193. PubMed PMID: 28250466; PubMed Central PMCID: PMCPMC5494038.

8. Rohou A, Grigorieff N. CTFFIND4: Fast and accurate defocus estimation from electron micrographs. J Struct Biol. 2015;192(2):216-21. Epub 2015/08/19. doi: 10.1016/j.jsb.2015.08.008. PubMed PMID: 26278980; PubMed Central PMCID: PMCPMC6760662.

9. Roseman AM. FindEM--a fast, efficient program for automatic selection of particles from electron micrographs. J Struct Biol. 2004;145(1-2):91-9. Epub 2004/04/07. doi: 10.1016/j.jsb.2003.11.007. PubMed PMID: 15065677.

10. Scheres SH. RELION: implementation of a Bayesian approach to cryo-EM structure determination. J Struct Biol. 2012;180(3):519-30. Epub 2012/09/25. doi: 10.1016/j.jsb.2012.09.006. PubMed PMID: 23000701; PubMed Central PMCID: PMCPMC3690530.

11. Zhang K. Gctf: Real-time CTF determination and correction. J Struct Biol. 2016;193(1):1-12. Epub 2015/11/26. doi: 10.1016/j.jsb.2015.11.003. PubMed PMID: 26592709; PubMed Central PMCID: PMCPMC4711343.

12. Punjani A, Rubinstein JL, Fleet DJ, Brubaker MA. cryoSPARC: algorithms for rapid unsupervised cryo-EM structure determination. Nat Methods. 2017;14(3):290-6. Epub 2017/02/07. doi: 10.1038/nmeth.4169. PubMed PMID: 28165473.

13. Wang RY, Song Y, Barad BA, Cheng Y, Fraser JS, DiMaio F. Automated structure refinement of macromolecular assemblies from cryo-EM maps using Rosetta. Elife. 2016;5. Epub 2016/09/27. doi: 10.7554/eLife.17219. PubMed PMID: 27669148; PubMed Central PMCID: PMCPMC5115868.

14. Emsley P, Cowtan K. Coot: model-building tools for molecular graphics. Acta Crystallogr D Biol Crystallogr. 2004;60(Pt 12 Pt 1):2126-32. Epub 2004/12/02. doi: 10.1107/S0907444904019158. PubMed PMID: 15572765.

15. Laskowski RA, MacArthur MW, Moss DS, Thornton JM. PROCHECK: a program to check the stereochemical quality of protein structures. Journal of Applied Crystallography. 1993;26:283-91.

16. Horn HW, Swope WC, Pitera JW, Madura JD, Dick TJ, Hura GL, et al. Development of an improved four-site water model for biomolecular simulations: TIP4P-Ew. J Chem Phys. 2004;120(20):9665-78. doi: 10.1063/1.1683075. PubMed PMID: 15267980.

17. D.A. Case RMB, D.S. Cerutti, T.E. Cheatham, III, T.A. Darden, R.E. Duke, T.J. Giese, H. Gohlke, A.W. Goetz, N. Homeyer, S. Izadi, P. Janowski, J. Kaus, A. Kovalenko, T.S. Lee, S. LeGrand, P. Li, C. Lin, T. Luchko, R. Luo, B. Madej, D. Mermelstein, K.M. Merz, G. Monard, H. Nguyen, H.T. Nguyen, I. Omelyan, A. Onufriev, D.R. Roe, A. Roitberg, C. Sagui, C.L. Simmerling, W.M. Botello-Smith, J. Swails, R.C. Walker, J. Wang, R.M. Wolf, X. Wu, L. Xiao and P.A. Kollman. AMBER. In: 2016, editor.: University of California, San Francisco; 2016.

18. Homeyer N, Horn AHC, Lanig H, Sticht H. AMBER force-field parameters for phosphorylated amino acids in different protonation states: phosphoserine, phosphothreonine, phosphotyrosine, and phosphohistidine. Journal of Molecular Modeling. 2006;12(3):281-9. doi: 10.1007/s00894-005-0028-4. PubMed PMID: WOS:000235756300004.

19. Hopkins CW, Le Grand S, Walker RC, Roitberg AE. Long-Time-Step Molecular Dynamics through Hydrogen Mass Repartitioning. J Chem Theory Comput. 2015;11(4):1864-74. doi: 10.1021/ct5010406. PubMed PMID: 26574392.

20. Le Grand S, Gotz AW, Walker RC. SPFP: Speed without compromise-A mixed precision model for GPU accelerated molecular dynamics simulations. Computer Physics Communications. 2013;184(2):374-80. doi: 10.1016/j.cpc.2012.09.022. PubMed PMID: WOS:000311661100012.

21. Salomon-Ferrer R, Gotz AW, Poole D, Le Grand S, Walker RC. Routine Microsecond Molecular Dynamics Simulations with AMBER on GPUs. 2. Explicit Solvent Particle Mesh Ewald. J Chem Theory Comput. 2013;9(9):3878-88. doi: 10.1021/ct400314y. PubMed PMID: 26592383.

22. Miao Y, Feher VA, McCammon JA. Gaussian Accelerated Molecular Dynamics: Unconstrained Enhanced Sampling and Free Energy Calculation. J Chem Theory Comput. 2015;11(8):3584-95. Epub 2015/08/25. doi: 10.1021/acs.jctc.5b00436. PubMed PMID: 26300708; PubMed Central PMCID: PMCPMC4535365.

23. Kikhney AG, Svergun DI. A practical guide to small angle X-ray scattering (SAXS) of flexible and intrinsically disordered proteins. FEBS Lett. 2015;589(19 Pt A):2570-7. doi: 10.1016/j.febslet.2015.08.027. PubMed PMID: 26320411.

24. Franke D, Petoukhov MV, Konarev PV, Panjkovich A, Tuukkanen A, Mertens HDT, et al. ATSAS 2.8: a comprehensive data analysis suite for small-angle scattering from macromolecular solutions. J Appl Crystallogr. 2017;50(Pt 4):1212-25. doi: 10.1107/S1600576717007786. PubMed PMID: 28808438; PubMed Central PMCID: PMC5541357.

25. Petoukhov MV, Franke D, Shkumatov AV, Tria G, Kikhney AG, Gajda M, et al. New developments in the ATSAS program package for small-angle scattering data analysis. J Appl Crystallogr. 2012;45(Pt 2):342-50. Epub 2012/04/01. doi: 10.1107/S0021889812007662. PubMed PMID: 25484842; PubMed Central PMCID: PMCPMC4233345.

26. Yang J, Zhang Y. I-TASSER server: new development for protein structure and function predictions. Nucleic Acids Res. 2015;43(W1):W174-81. Epub 2015/04/18. doi: 10.1093/nar/gkv342. PubMed PMID: 25883148; PubMed Central PMCID: PMCPMC4489253.

27. Svergun DI. Restoring Low Resolution Structure of Biological Macromolecules from Solution Scattering Using Simulated Annealing. Biophysical Journal. 1999;76(6):2879-86. doi: 10.1016/s0006-3495(99)77443-6.

28. Kozin MB, Svergun DI. Automated matching of high- and low-resolution structural models. Journal of Applied Crystallography. 2001;34(1):33-41. doi: 10.1107/s0021889800014126.

29. Saldanha SA, Kaler G, Cottam HB, Abagyan R, Taylor SS. A Novel Assay Principle for Modulators of Protein-Protein Interactions and its Application to non-ATP-Competitive Ligands Targeting Protein Kinase A. Analytical Chemistry. 2006;78(24):8265-572.

30. Lacroix E, Viguera AR, Serrano L. Elucidating the folding problem of alpha-helices: local motifs, long-range electrostatics, ionic-strength dependence and prediction of NMR parameters. J Mol Biol. 1998;284(1):173-91. Epub 1998/11/13. doi: 10.1006/jmbi.1998.2145. PubMed PMID: 9811549.

**SI Figures**

**S1 Fig. |** Negative stain EM confirms that RIIβ_2_J-C_2_ and WT RIIβ holoenzymes have similar architectures. (**A**) Representative micrograph for negatively-stained RIIβ_2_J-C_2_ holoenzyme. Example particles are shown in red boxes. (**B**) 2D class averages of RIIβ_2_J-C_2_ shown alongside projections of WT RIIβ holoenzyme crystal structure. The WT RIIβ_2_C_2_ crystal structure was filtered to 20Å. (**C**) Model and negatively stained EM density of RIIβ_2_J-C_2_ holoenzyme.

**S2 Fig. |** Cryo-EM structure of C2 symmetric RIIβ_2_J-C_2_ holoenzyme. (**A**) Representative tilted micrograph with the right half of the image showing picked particles. (**B**) Local CTF plot for micrograph in (A). Image generated using Appion and Gctf. (**C**) Representative 2D class averages. (**D**) 3D-FSC curve.

**S3 Fig. |** 3D classification of RIIβ_2_J-C_2_ holoenzyme. (**A**) 3D classification. (**B**) Local density map of RIIβ_2_J-C_2_ holoenzyme.

**S4 Fig. |** Temperature factor of RIIβ_2_C_2_ holoenzyme (PDB = 3TNP). Both the A-helix and the CNB-B domain reveal high temperature factor.

**S5 Fig. |** The comparison of RIα and RIIβ B/C/N-helices. (**A**) The B/C/N-helices of RIα and RIIβ have different hinge angles. (**B**) The B/C/N-helix of RIα have high helical propensity with a local minimum at Gly235^RIα^. In the RIIβ, helical propensity of B/C/N-helix is much lower with a break point at Tyr265^RIIβ^. (**C**) N-capping analysis of B/C/N-helix in the RIα and RIIβ. (**D**) C-capping analysis of B/C/N-helix in the RIα and RIIβ. The data used to make this figure can be found in S1 Data.

**S6 Fig.** | The overlaid of all states of each RIIβ-subunit protomer in RIIβ_2_J-C_2_ holoenzyme from MD simulations. Both CNB-domains show similar dynamics and have breakages at Tyr265. Residue Tyr265^RIIβ^ was shown as pink ball.

**S7 Fig.** | SAXS analyses of RIIβ_2_C_2_ and RIIβ_2_J-C_2_ holoenzymes. (**A**), (**B**) Guiner plots of RIIβ_2_C_2_ (A) and RIIβ_2_J-C_2_ holoenzymes (B). (**C**), (**D**) Kratky plots of RIIβ_2_C_2_ (C) and RIIβ_2_J-C_2_ (D) holoenzymes both show bell-shape peaks at low q and not converging to the q-axis at high q. (**E**), (**F**) Scattering plots at low q and the model fittings of RIIβ_2_C_2_ (E) and RIIβ_2_J-C_2_ (F) holoenzymes. The data used to make this figure can be found in S1 Data.

**S8 Fig. 8** | Cryo-EM structure of RIIβ_2_J-C_2_ holoenzyme reveals the general position of D/D domain. (**A**) The extra density near the residue Ile104^RIIβ^ locates at the central hole of RIIβ_2_J-C_2_ holoenzyme. Residues Ile104^RIIβ^ were labeled as yellow balls. (**B**) The extra density extends along the central hole to the same face as the CNB-B domains and J-domains. Residues Ile104^RIIβ^ were labeled as yellow balls.

**S9 Fig.** | Full coomassie blue staining SDS page of purified C-, J-C-, J-C(Δ1-13)-, J-C(Δ1-38)-, J-C(Δ1-54)-, and J-C(Δ1-69)-subunits. J-C(Δ1-69)-subunit is equivalent to C(Δ1-14)-subunit.

**S10 Fig.** | Displacement vector between CNB-A (Arg230^RIIβ^) and CNB-B (Arg359^RIIβ^) domains. Each pair of panels shows the two protomers (left and right) in the holoenzyme. Plots show θ(x), ϕ(y), and (d) distance in color. Each row is an independent simulation. Upper left panels are conventional MD, as shown in Figure 3 of the maintext and the other panels are from Gaussian accelerated MD. Full-length C (C-gamd) shows on average a muted asymmetry between protomer displacement vectors. Asymmetry (∆θ,∆ϕ,∆d) is measured as the average per frame difference of θ, ϕ, and d between protomers, calculated as: $\Delta X=100*\frac{\sum_{i,j}^{N} ({Xi}/\bar{Xi}- {Xj}/\bar{Xj})}{N}$, where i and j are protomers at matching frames.

**S11 Fig.** | Helical propensity analyses of C-subunit, J-C-subunit and C(1-14Δ). (**A**) Either fusing with J-domain or just simply deletion of first exon reduces the helical propensity of A-helix. (**B**) Capping propensity analysis of the A-helix. Ser14^C^ reveals a strong N-capping propensity. The data used to make this figure can be found in S1 Data.

**S12 Fig.** | Isoform-specific PKA structures and B/C/N-helix dynamics. PKA RIα and RIIβ undergo different conformational changes between holoenzyme form and cAMP-bound form.
