# Supplementary material for: Structural analyses of the PKA RIIβ holoenzyme containing the oncogenic DnaJB1-PKAc fusion protein reveal protomer asymmetry and fusion-induced allosteric perturbations in fibrolamellar hepatocellular carcinoma
Source: PLoS Biol. 2020 Dec 28;18(12):e3001018. doi: 10.1371/journal.pbio.3001018 (PMC7793292; doi:10.1371/journal.pbio.3001018)
Supplement: Attachment — Submitted filename: 20200913 Response to Reviewers.docx [file pbio.3001018.s017.docx]

Dear editors and reviewers,

We thank you for the comprehensive and positive review of our manuscript. The comments were very constructive, and we tried to address all of the concerns. We attach the revised manuscript as well as our specific responses to each reviewer. We also highlight the changes in the manuscript. We hope that the manuscript will now be considered suitable for publication in PLoS Biology.

Michael Cianfrocco

Susan Taylor

**Reviewers’ comments**

**Rev. 1:**
This is a nice study that uses a range of biophysical and structural techniques to probe the regulation mechanism of PKA. The study picks up from the group's previous crystallographic work which found a symmetric dimer of the RIIb2C2 holoenzyme, and using cryoEM for a fusion associated with fibrolamellar hepatocellular carcinoma (DnaJB1-PKAc) found that the holoenzyme might actually be asymmetric. Molecular dynamics shows that the fusion holoenzyme is relatively more stable than the wild-type, which displays increased asymmetry. A SAXS study is conducted which can be improved. Their enzymatic studies imply that asymmetry plays a role in regulation of activity, but as they rightly highlight in the discussion, the current study represents a first step towards understanding exactly what is occurring here. I have some specific comments that they might wish to address.

Comments
Figure 1A is not the clearest. They should illustrate which regions of PKA and DnaJB1 are fused together, and they might also wish to provide a schematic that describes the structures in the figure. Similarly, the asymmetry that they observe in their cryo map is perhaps not as easy to visualize in Figure 1D as it is in Fig 3B, perhaps they might consider promoting that panel to the main body.

This point is made by several reviewers and the figure has been modified accordingly. We agree that this is a significant improvement.

The hinge angles in Figure 2F and 2G are curiously accurate to 2 decimal places. I suspect the crystal structures provide an accuracy approximating to a few degrees, and would encourage the authors to take another look at these.

We have edited the Figure 2F and 2G as the reviewer suggested; the decimals in Figure 2F and 2G have been removed. We have also carefully compared and re-measured the angles between RIα and RIIβ, and the figure of RIα and RIIβ B/C/N-helices overlaid has been updated in Supplementary Figure 5A. It clearly shows the hinge angle differences between RIα and RIIβ. The difference might be subtle; however, we believe it is one of the critical reasons that lead to distinct cAMP-bound form structures of RIα and RIIβ (Supplementary Figure 12).

The SAXS studies can be significantly improved. These should be conducted over a range of concentrations to validate the differences observed. They should be presented in a manner described by Trewhella PMID: 28876235, and a full SAXS 'Table 1' presented. Figures 4E and F are uninformative without the envelopes. The spatial localization of the D/D domain from the SAXS analysis is unconvincing. Does the MW difference hold up in I-zero analysis?

We have updated Figure. 4 as Reviewer 1 suggested. We now include the *ab initio* shapes (or bead modeling) in the figure. Both the WT and fusion holoenzymes’ *ab initio* envelopes overlaid well with atomistic structure models that are derived from known crystal structures. We also updated the detailed description about ab initio models in the main text as well as in the experimental section.

With regard to sample quality, we used SEC-SAXS to remove any possible protein aggregations. In addition, the on-column auto R_g_ analysis revealed mono-distributed R_g_ values, indicating there is no concentration dependent aggregation/oligomerization. Moreover, both samples show no sign of aggregation according to the Guinier analysis at low scattering angles(q).

Unfortunately, SEC-SAXS limits the ability to determine protein concentrations (Acta Cryst. D., 2017, PWID 28876235). We don’t have accurate concentration estimation, therefore the molecular estimation from I(0) can be very challenging. However, the molecular weight estimation from both Porod volume and simply SDS-page analysis reveal similar molecular weights.

In this paper we were not using the SAXS analysis to confirm the molecular weight but were more concerned with showing that there was no aggregation. We carried out a previous detailed analysis of a deletion mutant of the RIIβ holoenzyme (JBC, 2014, PMID 25112875) where we rigorously addressed all of these issues.

It is unclear how many repeats have been conducted for the enzymatic assays in Fig 5, and what the error bars indicate.

We thank reviewers for pointing this out. We have included this information in the figure legend.

**Rev. 2:**
This study focuses on the structure and dynamics of PKA holoenzymes containing RIIbeta and DNAJB1-PKAc subunits, and includes a number of interesting observations. The authors utilise cryo-EM in one of the first (the first?) attempts at a high-resolution 3D reconstruction of a PKA holoenzyme using cryo-EM. Clearly, PKA is clearly not a straightforward sample for analysis by cryo-EM, and the masking approach employed to resolve the CNB-B and J domains is smart and creative. The structure reveals unexpected potentially asymmetric dynamics in the CNB-B/J domain regions of the holoenzyme. The study includes a very extensive investigation of IIbeta holoenzyme dynamics using MD simulations that supports the notion that dynamics are restricted to one RII protomer at a time. Another notable observation is electron density in the cryo-EM structure that helps to assign the position of the D/D domain relative to the rest of the holoenzyme. The study pulls together several complementary techniques (cryo-EM, SAXS, MD simulations, PKA activation assays), is well written, and the methods are described in detail.

I have a number of minor comments/questions:

1. In the abstract on lines 39-41, I was confused by the following statement: "properties of the RIIβ holoenzyme are also impaired by the J-domain; it is harder to activate with cAMP and the cooperativity is reduced". The data in Fig 5C show that the mutant holoenzyme is easier to activate with cAMP?

We thank the reviewer for pointing out this typographical error. It has been corrected It is **easier** to activate with cAMP and the cooperativity is reduced

2. Regarding the legend to figure 1. Can the authors clarify which PDB coordinate file is being shown in each panel? It isn't immediately apparent.

We appreciate the reviewer’s feedback. We have added the PDB information in the legend of Figure 1.

3. Regarding the rotations between panels in figure 1. The rotation operations between panels are unnecessarily complicated - if the left and middle views are switched then a simple 90 degree rotation between each panel will be sufficient.

This point is made by several reviewers and the figure has been modified accordingly. We think that the new figure is much clearer.

4. Regarding the cryo-EM processing (table 1 and methods). I notice that very large numbers of particles have been discarded during the processing (down from 642,843 to 69,605 for the C2 structure and 11,182 for the C1 structure), more so than in a typical cryo-EM reconstruction. Please can the authors comment on why this was necessary?

Although almost all of the particles were in a compact globular conformation, we believe that the intrinsic flexibility of both the core and CNB-B domains required us to enrich only for particles in a particular conformation. We agree that the cryoEM data provides only a small snapshot of the structural dynamics, which is why we have included the SAXS and molecular dynamics to help construct a more complete understanding of the PKA holoenzyme. We have included this point in discussion section of the main text to make sure the reader also appreciates this consideration (line 374-379).

5. Regarding how this study relates to other structural studies of PKA that used EM. The intro does not refer to other EM studies. I am aware of one paper from the Scott/Gonen labs that used negative stain EM (E-life, 2013, PMID 24192038). This study focused on type II PKA-AKAP18gamma complexes, and the authors reported several extended conformations in which the two RII-C protomers were separated by some distance. This study does not include any cryo-EM experiments, however, and does not attempt to quantify the abundance of different conformational states. Did the authors of this study observe any extended conformations of PKA?

We thank the reviewer for bringing up the Scott/Gonen lab’s work on the RIIα holoenzyme. We have now referenced this negative staining EM work and also highlighted that ours is the first cryoEM structure of a PKA holoenzyme. The differences between RIIα and RIIβ holoenzymes is also very important point that we did not stress sufficiently. We have now pointed this out in the introduction, in the results, and in the discussion.

We have shown in our previous studies that both cAMP-bound form RIIα_2_ and RIIβ_2_ are fully extended. The RIIβ, however, becomes compact following addition of the C-subunit, which is in contrast to the RIIα holoenzyme which remains extended (JMB, 2004, PMID 15046986). We also showed in that paper in a linker swap experiment that the difference is mediated by the linker region that joins the D/D domain to the inhibitor site. The RIIβ holoenzyme becomes more elongated by switching linker region with RIIα (JMB, 2004, PMID 15046986). Moreover, the RIIβ holoenzyme interface is RIIβ-specific, which is not conserved in any other R-subunit isoforms (Science, 2012, PMID 22323819). Finally, RIIα and RIIβ also have distinct cAMP sensitivity and Hill slope which can reflect their different quaternary structures (Science, 2012, PMID 22323819). We believe these are fundamental differences between RIIα and RIIβ. The importance of this interface is also captured in a subsequent paper where the linker region preceding the Inhibitor Site is trapped in a conformation that orients it towards the center hole (JMB, 2009, PMID 19748511).

In our cryoEM studies, the majority of the particles show compact conformations which is consistent with our wild-type RIIβ holoenzyme crystal structure.

6. Regarding the novelty of the cryo-EM reconstruction. There do not appear to be any other published 3D reconstructions of PKA using cryo-EM. Is this correct? It would be helpful to include an additional statement in the intro or discussion explaning how this work relates to other EM investigations of PKA.

This is indeed the first cryoEM structure of any PKA holoenzyme and the reviewer is correct that it is distinct from the preliminary low resolution negative staining data that was reported earlier for the RIIα holoenzyme. We now point this out in the abstract and in the introduction and comment in the discussion on the differences as pointed out in our response to your previous question.

7. Regarding the issue of asymmetry as detected in the cryo-EM reconstruction. In the initial C2 averaged structure (using ~ 69K particles), the CNB-B and J domains are not visible. By applying a mask and utilizing only a subset of the particles (~11K in the final refined structure), the authors are able to resolve the CNB-B and J domain in one half of the holoenzyme. They conclude that this shows asymmetry in the holoenzyme with one RII-JC promoter more ordered than the other. Is it possible that this apparent asymmetry has arisen due to selective sampling of the particle images? Ie the same conformation is in fact present at the same frequency in both protomers (ie at a frequency of about 1 in 7) such that this conformation can only be resolved in the protomer on which the averaging is focused? Please can the authors comment on this possibility? A statement should be included in the main text at least considering this possibility. Perhaps it is also possible to try to further classify the 11,182 particles to look for a subset that show the same ordered conformation in both protomers?

Yes, we agree that seeing the CNB-B and J domain for one protomer does not provide information on the other protomer. A larger dataset is needed for us to explore the occupancy and coordination between these two protomers considering how few particles made it to the final reconstruction. We tried to explore whether we could classify the data further for the 11,182 particles but there were too few particles to see differences in the classes. We also include a statement about this to highlight this point to the reader (line 374-379).

8. On lines 444-445. "However, any differences in dynamics are masked in these holoenzyme crystal structures; they are simply averaged". Is it not more likely that crystal packing prevents these movements vs averaged out during data processing?

We agree with reviewer’s comment and changed this sentence in the manuscript. “However, any differences in dynamics, in particular of the CNB-B domains, are masked in these holoenzyme crystal structures most likely by crystal packing.” (line 475-477)

9. In supplementary figure 12, the legend refers to coomassie-stained gels that are absent in the figure (they appear to have been moved to figure 5A). Please can the authors correct this.

We thank the reviewer for pointing out this error. We have corrected this mistake in the revised manuscript.

**Rev. 3:**
This interesting paper uses a combination of CryoEM and MD models to examine the structure of DNAJB1-PKACA (J-C) fusion protein: RIIβ tetrameric holoenzyme. The J-C fusion is caused by a genomic translocation which drives a rare form of liver cancer and is worthy of study. The data presented are interesting and shed light on both the impact of fusion (biochemical and structural) and also potentially on the behaviour of the endogenous PKA RIIβ holoenzyme. Unlike their previous PKA RIIβ holoenzyme crystal structure the D/D domain is visible in the CryoEM, which adds value to the new structure. Also they report intrinsic asymmetry with two J-C protomers in the tetramer and suggest this may be important for regulation of the holoenzyme. There are however a couple of issues which should be addressed to clarify interpretation, particularly with regard to extrapolating findings to the endogenous PKA complex.

Major Issue:
1. Fusion vs truncation. The paper focuses on the structure of the J-C fusion which is valuable. Comparisons are made with the C subunit alone, but the studies often compare the J-C fusion with the C subunit without the N-terminal 14aas (as they are deleted in the fusion). In particular, the MD dynamics do not include this 14aa section of the C subunit. This is important as it is not clear whether many of the observations made are because of deletion of the 14aa segment OR because of fusion with the J-subunit. Linked to this, the authors often discuss how this holoenzyme structure - and in particularly asymmetry - could be crucial to holoenzyme function. It should perhaps be made clearer that the current structure has a mutant catalytic subunit, which may of course behave differently to the native form with no fusion and with no truncation. It is understood that generating a Cryo-EM structure of the native holoenzyme for comparison may be a huge undertaking. However, as a minimum, the MD analysis could be conducted on the C-subunit with the inclusion of the N-terminal 14 aas. Perhaps with the FL-C subunit, the complex will behave differently.

This comment is similar to Reviewer 4’s comment and we have addressed this comment (…the MD dynamics do not include this 14aa section of the C subunit… It should perhaps be made clearer that the current structure has a mutant catalytic subunit, which may of course behave differently to the native form with no fusion and with no truncation…) below in response to Reviewer 4 - by making it more clear what was run in the conventional MD.

RE: “However, as a minimum, the MD analysis could be conducted on the C-subunit with the inclusion of the N-terminal 14 aas. Perhaps with the FL-C subunit, the complex will behave differently.”

We originally ran the conventional MD on the RIIβ_2_C(Δ1-14)_2_ and RIIβ_2_J-C_2_, 1) because the first 14 residues of the C-subunit are typically disordered in crystal structures, and 2) the J-domain fusion replaces these first 14 residues. After observing that the length of the N-terminus may have an impact on asymmetric dynamics, we then repeated the simulations with these two constructs and also full-length wt RIIβ_2_C_2_ using accelerated MD. We used accelerated MD to more robustly sample conformational space to ensure the original results were not reflecting local minima. Results were consistent between simulations: the length as well of composition of the N-terminus of the C-subunit had an impact on the asymmetric dynamics.

We have added more clarifying language to lines 230-232: “Together, this implies that the length of the N-terminus of the C-subunit, whether longer as in the J-C-subunit or shorter as in the C-subunit used here, may have a differential impact on the observed asymmetric dynamics. ”

And then also emphasized more clearly the reason we ran the accelerated MD on lines 316-319:

“To investigate how exon 1 of the C-subunit influence the asymmetric dynamics and cAMP activation of the RIIβ holoenzyme, Gaussian accelerated MD simulations (GaMD) on RIIβ_2_C_2_, RIIβ_2_J-C_2_, and RIIβ_2_C(Δ1-14)_2_ were carried out. Specifically, we ask whether it is the J-domain or the absence of the residues 1-14 that account for the differences.”

2. This main point is particularly important given the biochemistry in Figure 5 , showing that deletion of the 14aa N-terminus has a similar impact on cAMP binding to addition of the J-domain. This implies that the J-domain may be dispensable for the alteration to biochemical function. Related to this, referring to the CΔ1-14 as RIIβ2J-C(Δ1-69)2 is a bit misleading (it contains none of the J domain). Although the fact that they are the same thing is made clear, through the text it should either be referred to as CΔ1-14 or given both descriptions.

Both figures and the manuscript have been modified based on the reviewer’s suggestions.

3. In the text, differences in the B/C/N helices between protomers in the Cryo structure are discussed - could these differences be made more clear in a figure as they are somewhat lost in the images of the whole structure? (Lines 133 and 134)

This point is made by several reviewers and the figure has been modified accordingly.

4. The authors state the holoenzyme orients in a particular way on the cryo EM grid. Might attachment to the grid affect the dynamics of the two distinct protomers, direct the asymmetry or impact on the described loss of density in some regions? I acknowledge that the MD analysis provides an orthogonal approach.

We thank the reviewer for mentioning this – yes, as with all structural techniques, the way in which the structure is interrogated may affect aspects such as the structural dynamics. We agree that this is a caveat and will make sure to emphasize this consideration to the reader (line 374-379).

5. Line 83: "To understand the pathological consequences of FL-HCC, we used single particle cryo-electron microscopy (cryo-EM) to determine a structure of the RIIβ holoenzyme formed with J-C." This sentence is a bit misleading. Do the authors mean to understand the role of the J-C fusion in FL-HCC pathology?

We appreciate the reviewer raising this point. We have updated the manuscript “To provide mechanistic insight into the functional consequences of the DnaJB1-PKAc fusion protein that might allow us to better understand how it is a driver of FL-HCC, we used single particle cryo-electron microscopy (cryo-EM) to determine a structure of the RIIβ holoenzyme formed with J-C.”

6. Minor: In the introduction, the authors note that in FL-HCC, RIa is upregulated and RIIb is downregulated. This could imply that the stability or biochemistry of the complex may result in compensatory changes in subunit expression. This may also suggest that the J-C is preferentially complexed with the RI subunit in tumours (rather than as part of the RIIb structure presented here). Could this be commented on? Are there data on the stability or half life of distinct J-C holoenzymes?

We appreciate the reviewer’s suggestion. These are possible but nothing is really known yet about the composition of each holoenzyme in cells. We agree that this is a very important question, but it is not even known which of which R-subunits is preferred by the J-C subunit. In vitro measurements showed that both the J-C-subunit and C-subunit reveal similar binding affinity to RIα or RIIβ subunit (reference 4 in the manuscript). We just know that both holoenzymes are stable. We agree that this needs to be addressed but this is a separate question.

Even though we do not fully understand the stability of J-C holoenzymes in vivo, a more detail effect of this oncogenic protein on RIα has been discussed in our latest publication (reference 54 in the manuscript). The fusion protein disrupts RIα-phase separation bodies formation. This phenotype could be the reason for the compensatory effect of up-regulation of RIα; however, more studies need to be conducted to prove this assumption.

Linked to this, above, interaction between J and CNB-B, results in apparent CNB-B stabilisation. Given the origin of this chimeric protein, such interactions are clearly not evolved but rather are interactions driven by their chance proximity (resulting from the in frame translocation). This is perhaps not given clear consideration in the text although the differential with RIa is covered.

We thank for the reviewer pointing this out. We have modified the text based on the reviewer’s suggestion. We think that the description now is much clearer.

**Rev. 4: William Rice – note that this reviewer has waived anonymity**
Introduction
This paper examines the structure of the fusion protein J-C subunit bound to RIIβ. The JC subunit is created when the J domain of Dna JB1 replaces exon 1 of cAMP dependent protein kinase (PKA), and it is a potent driver of Fibrolamellar heptatocellular carcinoma. The authors used a combination cryo-EM, SAXS, and molecular dynamics to produce moderate resolution (6-8 Å) structures of the holoenzyme and examine its structural dynamics. Although they do not reach high resolution, they were able to identify various domains of the protein and compare it with a prior crystal structure of the wild-type construct. This will be of interest to the many groups on the field studying PKA activation and cancers related to the overactivation of this protein. The dynamical results will inspire experiments to more fully understand the activation and mechanism of this complex. I believe it is of enough general interest to publish in PLOS Biology.

Summary of Results
The holoenzyme has stoichiometry RIIβ2J-C2. The cryo-EM structure of the holenzyme itself was only of moderate resolution, 6.5 Å, from 69,605 particles with C2 symmetry applied. Much of the mass was missing, including the J domains, cyclic nucleotide B (CNB-B) domains, dimerization and docking (D/D) domains were missing. Further analysis using 3D classification and removing C2 symmetry followed by focused classification revealed a subset of 11,182 particles which did show the J and CNB-B domains in one of the subunits of the holoenzyme, at 7.5 Å resolution. The authors conclude that these regions must be flexible, there must be asymmetry between the protomers in these domains, and presumably both are important for the function of this protein. Molecular models were made from the maps using previously published PDB coordinates and homologous model structure prediction, followed by real space refinement.

Molecular dynamic simulations of RIIβ with wt C subunits indicated that the C subunit was stable, as was the CNB-A domain of the RIIβ in one of the protomers. In contrast, the CNB-B in the other protomer appeared to have a large amount of flexibility, in agreement with the asymmetry seen in the cryo-EM map of RIIβ2J-C2. The pivot point appears to be at Tyr265, located in the BCN helix of RIIβ. The same simulations performed on the RIIβ2J-C2 holoenzyme showed that the CNB-B subunits of both protomers had a similar amount of flexibility, and both were more stable than the flexible CNB-B subunit of the wt form. Both J domains were overall more flexible than the remainder of the C domain. They hypothesize that the J domains are coupled to the CNB-B domains, due to their apparent close proximity in the cryo-EM map. Analysis of the flexibility between the CNB-A and CNB-B domains also showed greater flexibility in one protomer than the other for both wt and RIIβ2J-C2 isoforms, though flexible wt protomer moved more than the flexible RIIβ2J-C2 protomer. Further MD simulations using GaMD measured the solvent accessible surface of RIIβ2C2, RIIβ2J-C2, and RIIβ2J-C(Δ1-69)2. Both constructs had more solvent accessible area than the wild type, and distance measurements between two atoms in the PBC pocket also showed more open states than seen in the wt complex. There was more asymmetry observed between the protomers than seen in the wt complex.

SAXS analysis of wt and RIIβ2J-C2 holoenzymes showed similar results for both, with indications of stably folded domains and a high degree of flexibility. The RIIβ2J-C2 isoform showed a slightly higher radius of gyration and a higher deviation of predicted versus calculated molecular weight. Finally, they measured activation of wt and fusion holoenzymes by cAMP, as well as successive deletion mutants of the RIIβ2J-C2 where successive N-terminal helices of the J domain were removed. Apart from the wt, all fusion holoenzymes showed a higher affinity for cAMP and lower cooperativity between subunits.

Based on these results, and prior data in the literature, a model for J-domain activation is presented.

Review
This is a significant paper in that the moderate resolution structure of an ocogenic fusion protein was solved. Likely due to its inherent flexibility, it only went to 6.5 Å but further processing revealed a previously unseen asymmetry in the protomer. This led to molecular dynamics simulations, which also predict an asymmetry on the protomer. The structure came from 1,129 micrographs, which is a moderate dataset nowadays. Adding more images would likely improve the resolution, but given the flexibilities involved, likely not enough to significantly alter the results. The most significant part of the structural interpretation which seems to not be on solid ground is the D/D location, discussed below:

D/D Domain
Results Lines 262-273: The positioning of the D/D domain due to the “extra density” in the map seems speculative and should be in the Discussion part of the paper. Although there is other experimental evidence such as crosslinking which points to its localization, the extra density seen in the map is at best very low resolution and it is hard to justify using it to place atoms. Considering the moderate resolution of the rest of the map, fitting anything in this poorly resolved density seems unjustified. There is no evidence in the cryo-EM map to place the D/D domain in close proximity to the CNB-B and J-domains. This could be moved to the discussion section in interpreting the map. The extra density could be an artifact due to low resolution, though it could be the linking region. As shown in Supplemental Figure 9, it does not seem to match two unstructured loops. The cutoff value for the density used in Figure 9 also does not match the J/A helix well (left side of Figure B) but there is no extra protein proposed to fill this region.

Discussion line 337: The cryo-EM map does not give the general localization of the D/D domain. It is not seen, there is only some undefined density above the proposed location which the authors propose to be a linker to this domain. This should not be a primary conclusion of this paper.

Discussion line 381: The results do not show that the linker joining the D/D domain weaves through the hole in the dimer. As mentioned above, this is conjectured but not shown by the observed extra density. The conclusion is made too strongly given the data at hand. It is OK to hypothesize the location but the maps do not definitively find it.

In short, the conclusions about this domain should be made much less strong. Stronger evidence would be required to justify these conclusions. This could possibly come from further analysis/classification of the existing data, but would likely need more data to be added, if it could be resolved at all. I do not recommend more data, unless it is already available or the authors are willing, but in the current form the conclusions about the domain should be lowered.

We thank the reviewer for pointing out this concern. The paragraph of the D/D domain position justification from cryoEM map has been left in the results section, but the rest of this argument is now moved to the discussion as the reviewer suggested. We also modified the discussion of D/D domain position. We agree that we can’t place D/D domain into cryoEM density unambiguously; however, our SAXS data, combined with our previous studies, can still give us some clues about the general position of the D/D domain, and all of these lines of evidence are consistent. Our observations are also consistent with the cross-linking data from Gold’s lab.

In the revised manuscript, we also point out that this structure is very different from the negative staining results for RIIα. Finally, we point out that the conformation of the RIIβ holoenzyme will be significantly influenced by proximity to membranes. Elucidating these differences will be one of the important future challenges.

The extra density in the Supplementary Fig. 9 (now Supplementary Fig. 8) has again been carefully examined. We agree that the density in Supplementary Fig. 9 (now Supplementary Fig. 8) does not perfectly match the J-domain and A-helix. However, the density can still cover most part of the J-C-subunit N-terminus, especially the J-domain (Fig. 3C). The low density contour level also reflects the dynamic feature of those domains. From our analyses in this paper and previous studies (reference 11 and 12 in the manuscript), the MD analyses, biochemistry, SAXS, and crystal structures all reveal the flexible properties of J-domain and A-helix. The structure here, consistent with our MD simulations, perfectly reveals the flexible feature and domain properties of RIIβ_2_J-C_2_ holoenzyme.

Relation between J and CNB domains
Results line 278-279: The 7.5 Å C1 structure shows that the J and CNB domains are close by each other. However the MD simulations presented do not seem to indicate that the J domain moves in a correlated way with its adjacent CNB-B domain. I do not see how panels 2B,F, and G show such a correlation.

We thank the reviewer for pointing out this concern. We have modified the manuscript to better describe the communications between the J-domain and CNB-B domain.

Asymmetry between protomers
Results Line 314-315: The GaMD simulations seem to show less asymmetry in the wt holoenzyme and more in the RIIβ2J-C2 construct. This seems to be the opposite from the other MD simulations in Figure 2, A-E and Figure 3E and F, as well as Supplemental Figure 7. However here the authors state that the wt protomers are tightly coupled, the exact opposite of what had been said previously. These differing results should be discussed.

We thank the reviewer for pointing out this seeming discrepancy. We believe it is due to our original description of the two simulations lacking clarity. We have added more descriptive language to this section at line 156-159: “The first 14 residues of the C-subunit, which are typically disordered in our crystal structures and are replaced by the J-domain in the J-C fusion, are not included in these simulations; this construct is referred to as C(Δ1-14). The last 23 residues of the RIIβ subunit as well as the linker region and the D/D domain (residues 1-103) are also not included.”

We also will use the same nomenclature for constructs here as later in the text: substituting for the conventional MD RIIβ_2_C_2_ with RIIβ_2_C(Δ1-14)_2_.

The large difference observed in conventional MD between Figure 2A-E and Figure 3E-F is mainly because these show the C(Δ1-14) construct (not full-length) and the J-C construct, respectively. We then followed up these simulations with accelerated MD on full-length C, C(Δ1-14) , and J-C: to 1) support our conventional MD showing the length of the N-terminus has an impact on RIIβ:C dynamics and 2) to more robustly sample conformational space (to ensure what we observed by conventional MD wasn’t due to local minima).

Figures
Figure 1: The choice of white as the map density makes it quite hard to see, particularly since the C domain model is also white. If possible it would be good to add some more contrast. It is particularly hard to see how well the density fits the J/A helix in panels D and E.

This point is made by several reviewers and the figure has been modified accordingly.

Supplementary Figure 12: The figure legend describes A,B, and C, but only A and B are shown, which correspond to B and C in the text. A, described in the text, seems to have been replaced by Supplementary Figure 10

We thank the reviewer for pointing out this mistake. We have corrected this in our revised manuscript*.*

Other
Line 142: Seems to be a word missing after “crystal” (symmetry perhaps?)
Lines 155-157: Mentioning of missing 14 residues is redundant (already explained in prior paragraph)
Line 314: “consistence” should be “consistent”
Line 472: no all-caps on “every”.

We have corrected these errors in our revised manuscript.

Recommendation
I recommend publication upon revision of the text. I do not recommend any new data be collected, provided the conclusions about the D/D location are weakened. The current conclusions would require stronger evidence in the cryo-EM map. The methodology seems clearly presented in the Supplemental methods, and the Supplementary Figures all support the paper and are presented well. The data have been submitted to the appropriate repositories.
